# Water-insensitive NIR-I-to-NIR-I down-shifting nanoparticles enable stable biomarker detection at low power thresholds in opaque aqueous environments

**DOI:** 10.1038/s41377-025-01882-2

**Published:** 2025-07-03

**Authors:** Dongkyu Kang, Suyeon Kim, Yeongchang Goh, Minseo Kim, Sun-Hak Lee, Jung-Hoon Kwon, Sang Hwan Nam, Joonseok Lee

**Affiliations:** 1 Department of Chemistry, Hanyang University, Seoul, 04763 Republic of Korea; 2 Laboratory of Nanoscopic Imaging & Spectroscopy Analysis, Chemical Analysis Center, Korea Research Institute of Chemical Technology, Daejeon, 34114 Republic of Korea; 3 Avian Disease Laboratory, College of Veterinary Medicine, Konkuk University, 120 Neungdong-ro, Gwangjin-gu, Seoul, 143-701 Republic of Korea; 4 Laboratory of Veterinary Microbiology, College of Veterinary Medicine, Kyungpook National University, 80 Daehak-ro, Daegu, 41566 Republic of Korea; 5 Research Institute for Convergence of Basic Sciences, Hanyang University, Seoul, 04763 Republic of Korea

**Keywords:** Nanoparticles, Quantum dots, Imaging and sensing

## Abstract

Luminescence quenching in aqueous environments poses a challenge for practical applications. Lanthanide-doped up-conversion nanoparticles (UCNPs), representative of near-infrared (NIR)-emitting phosphors, typically utilize Yb^3+^ ions as sensitizers, requiring 980 nm light. This wavelength coincides with the transitions of water molecules, interfering with population dynamics, and continuous irradiation causes unintended heating. Although Nd^3+^ ions, which absorb at 800 nm, serve as alternative sensitizers, their practical use is limited by low quantum yield (Q.Y.). In this study, we developed water-insensitive down-shifting nanoparticles (WINPs) functioning within the NIR-I range (700–900 nm) to avoid water interference. Characterization through single-particle-level spectroscopy demonstrated water-insensitive properties, with identical powers density and lifetime profiles under both dry and water conditions. The WINPs achieved a high Q.Y. of 22.1 ± 0.9%, allowing operation at a detection limit power 15-fold lower than UCNPs, effectively eliminating background noise and enhancing overall performance. To assess diagnostic potential, we validated WINP-based lateral flow immunoassay (LFA) for detecting avian influenza viruses (AIVs) in 65 opaque clinical samples, achieving 100% sensitivity and an area under the curve (AUC) of 1.000 at only 100 mW cm^−2^. These findings highlight the potential of WINPs as water-insensitive NIR phosphors that can operate at low power, even in water-rich environments.

## Abstract (web summary)

Water-insensitive NIR-I-to-NIR-I down-shifting nanoparticles (WINPs) enable low-power, high-sensitivity virus detection in opaque samples by overcoming water-induced quenching and providing a more stable signal than conventional upconversion nanoparticles.

## Graphical abstract

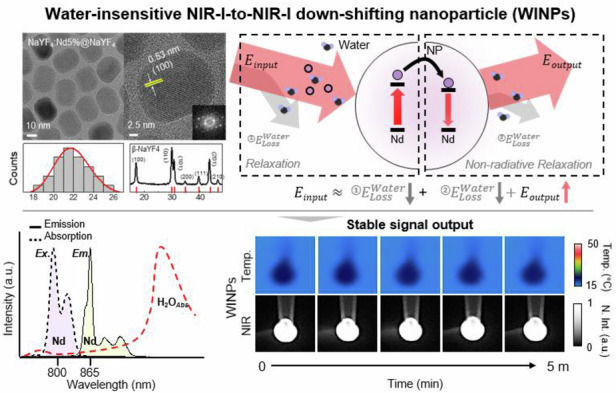

## Introduction

Near-infrared (NIR)-based optical technologies have emerged as promising tools in biomedical research because they enable real-time and highly sensitive monitoring of biomolecular interactions and high-resolution imaging in biological environments^1–6^. The so-called “biological window,” which encompasses the NIR-I (700–900 nm) and NIR-II (1000–1700 nm) ranges, offers numerous advantages, such as low auto-fluorescence and reduced scattering, facilitating deeper tissue penetration and yielding a high signal-to-background ratio (S/B) in biological contexts^7–9^. Lanthanide-doped up-conversion nanoparticles (UCNPs) are anti-Stokes emitters capable of NIR-to-NIR conversion^10–12^. Typically, NIR-emitting UCNPs are composed of ytterbium (Yb^3+^) ions as sensitizers, which absorb infrared radiation at 980 nm and non-radiatively transfer their excitation to doped activator ions, such as thulium (Tm^3+^) ions, resulting in NIR emission at 800 nm (980-to-800 nm). Recent studies have reported the advantages of NIR-emitting UCNP-based optical sensors as powerful tools for developing biological applications with highly sensitive detection capability^13–15^. However, the absorption peak of Yb^3+^ ions at 980 nm significantly overlaps that of overtones of water molecules, leading to attenuation and unwanted heating effects in water-rich biological environments^16–18^. To address these issues, neodymium (Nd^3+^) ions, which absorb infrared radiation at 800 nm, have been used as alternative sensitizers. This substitution reduced the absorption coefficient of water from 0.48 cm^−1^ at 980 nm to 0.02 cm^−1^ at 800 nm, a 24-fold reduction that effectively minimizes heating effects^19^. Despite these improvements, Nd-sensitized up-conversion (UC) still suffers from low quantum yield (Q.Y. <1%), necessitating the use of high-power densities, which also increases the risk of photo-damage to cells and target analytes. Moreover, the need for a high power density places a strain on laser stability, potentially shortening the lifespan of the equipment and posing challenges for the commercialization of diagnostic tools owing to energy inefficiency and increased costs. Furthermore, their UC emission spectra are confined to the visible range (400–700 nm); moreover, their detection in the NIR-II region is also restricted owing to their reliance on expensive InGaAs detectors and complex cooling systems.

**Scheme 1:**
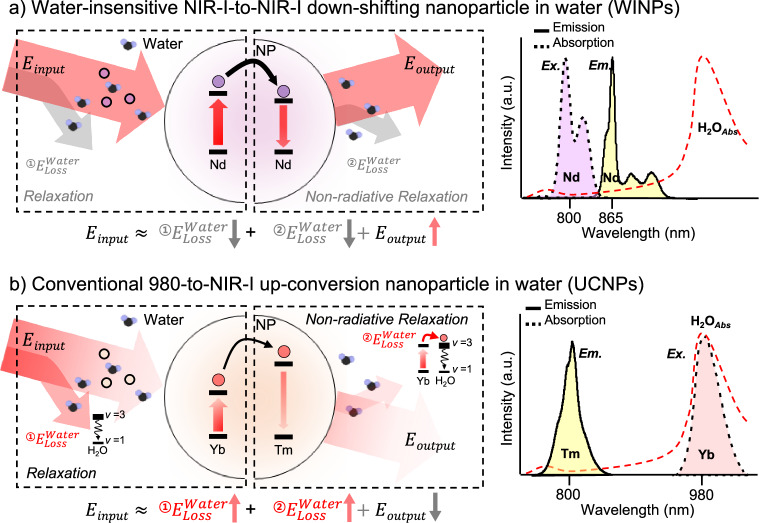
Schematic illustrations of NIR energy shifting process in water. **a** Water-insensitive NIR-I-to-NIR-I down-shifting nanoparticle (WINPs, NaYF_4_:Nd@NaYF_4_) and **b** conventional 980-to-NIR-I up-conversion nanoparticle (UCNPs, NaYF_4_:Yb,Tm@NaYF_4_)

Here, we introduce water-insensitive nanoparticles (WINPs) designed to function entirely in the NIR-I region. Scheme 1a illustrates the NIR-I-to-NIR-I down-shifting (DS) process of WINPs in water, in contrast to the UC process (Scheme 1b). The WINPs were finely optimized at NaYF_4_:5%Nd@NaYF_4_ to efficiently convert light absorbed at 800 nm into emission at 865 nm through the DS process. Single-particle-level characterization demonstrated water insensitivity and maintained a consistent detection limit power and lifetime profile in both dry and aqueous environments. Compared with UCNPs, WINPs achieved a 15-fold lower detection limit power in water, highlighting the superior efficiency of the DS. Under irradiation at 800 nm, the thermal effects were minimal, ensuring stable signal output from the WINPs, even over durations exceeding 5 min. Consequently, we applied this approach to validate water-independent, highly sensitive detection using a lateral flow immunoassay (LFA). Avian influenza viruses (AIVs) were chosen as target analytes because they are typically detected in opaque avian stools and cloacal swab samples^20–22^, making this a suitable test for evaluating the performance of WINPs in aqueous samples under low-power irradiation. The WINP-based LFA demonstrated high diagnostic accuracy and reliability when tested against 65 opaque clinical samples, achieving a sensitivity of 100% and an area under the curve (AUC) of 1.000, even under 100 mW cm^−2^ irradiation. Therefore, the developed WINPs offer considerable potential for use as water-independent biosensors in water-rich biological contexts that operate effectively at low power densities.

## Results

### Characterization of NIR-I-to-NIR-I down-shifting WINPs

**Fig. 1:**
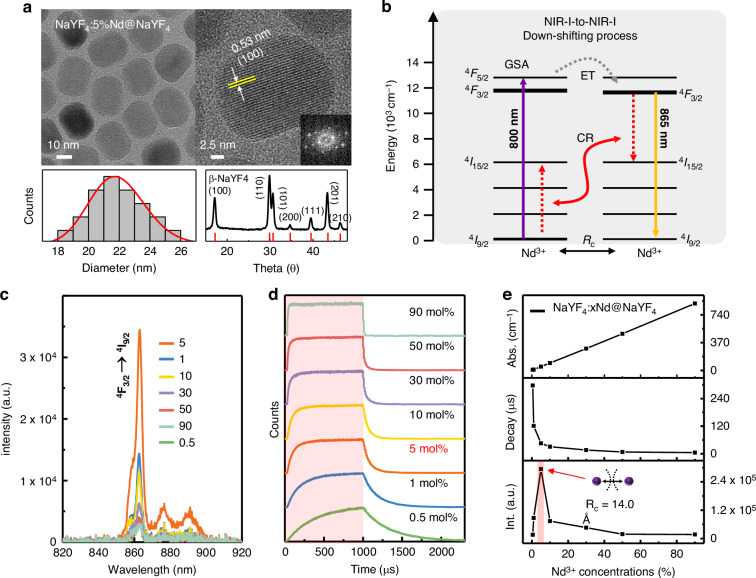
Characterizations of water-insensitive nanoparticles (WINPs). **a** TEM images of WINPs (top left), high-resolution TEM image (top right), and corresponding Fourier transform diffraction pattern (inset). Size distribution histogram (bottom left) and X-ray diffraction (XRD) patterns (bottom right). **b** Schematic illustration of the NIR-I-to-NIR-I down-shifting process in WINPs under 800 nm excitation. **c** NIR-I emission spectrum of NaYF_4_:*x*%Nd@NaYF_4_ (*x*: 0.5–90 mol%) under c.w. 800 nm excitation with a power density of 0.1 W cm^−2^. **d** Time-dependent emission profiles of NaYF_4_:*x* %Nd@NaYF_4_ (*x*: 0.5–90 mol%) at the transition of ^4^F_3/2_ → ^4^I_9/2_ under 1 ms pulsed laser excitation. **e** Characterizations of as-synthesized nanoparticles; (top) absorption coefficient of NaYF_4_:*x*%Nd@NaYF_4_ at around 800 nm wavelength, (middle) lifetime decay time, and (bottom) NIR emission intensities as a function of Nd^3+^ concentrations (0.5–90 mol%). (a.u. arbitrary units)

To optimize the WINPs, we synthesized a series of NaYF_4_:*x*%Nd@NaYF_4_ (*x* mol%, where *x* ranges from 0.5 to 90) nanoparticles via a thermal decomposition method^23^. The core–shell nanoparticles were synthesized by directly growing a NaYF_4_ inert shell on pre-synthesized core nanoparticles (Fig. S1). This process enhances photoluminescence (PL) through surface passivation, which minimizes surface defects and thereby improves the optical performance^24^. As shown in Fig. S1, doping of Nd^3+^ ions into NaYF_4_:*x*%Nd@NaYF_4_ does not have an observable effect on morphology and size. The as-synthesized WINPs (NaYF_4_:5%Nd@NaYF_4_) exhibit a consistent size distribution with an average diameter of approximately 21.9 ± 1.8 nm. High-resolution transmission electron microscopy (TEM) image reveals distinct lattice fringes with a *d*-spacing of 0.53 nm, corresponding to the interplanar distance of (100) crystalline planes (Fig. 1a). The Fourier transform diffraction pattern and the selected area electron diffraction (SAED) pattern indicated well-defined crystallinity. Additionally, X-ray diffraction (XRD) patterns confirmed the formation of the β-phase of NaYF_4_, which is known for its low phonon energy, reducing nonradiative losses and enabling efficient luminescence. This property makes it an effective host for facilitating specific energy transfer pathways between lanthanide ions, thereby enhancing overall photoluminescence efficiency^25^.

Figure 1b illustrates the NIR-I-to-NIR-I DS process between Nd^3+^ ions under 800 nm irradiation. Upon absorption in their ground states (ground state absorption, GSA), the ions undergo nonradiative relaxation and quickly accumulate in the ^4^F_3/2_ state. Typical PL emission bands of Nd, peaking at 865 nm corresponding to the ^4^F_3/2_ → ^4^I_9/2_ transition, were observed (Fig. 1b, c)^26–28^. In this state, optimizing the concentration of Nd^3+^ ions is crucial for achieving an ideal spacing between the ions. This optimization is essential because decreasing the spacing between Nd^3+^ ions leads to cross-relaxation, resulting in non-radiative relaxation and a subsequent decrease in the radiative PL intensity^29^.

To better understand the temporal characteristics of ionic interactions, we measured time-dependent PL lifetime profiles under a pulsed laser with a width of 1 ms^30^. Figure 1d shows that the lifetime decay in the ^4^F_3/2_ state significantly accelerates, decreasing from 297.1 to 58.2 μs as the doping concentration increases up to 5%. Generally, the PL intensity increased monotonically with increasing concentrations of Nd^3+^ ions (Fig. 1e). However, beyond a critical doping concentration, the decreased interionic distance results in stronger ion-ion interactions that enhanced non-radiative relaxation, a phenomenon known as concentration quenching, ultimately leading to a reduction in PL intensities^31–33^. According to Blasse’s equation, the critical distance (*R*_c_) between Nd^3+^ ions at 5% concentration was calculated as 14.0 Å^34,35^ (Fig. S2). As a comparison, we synthesized NaYF_4_:48%Yb,2%Tm@NaYF_4_ nanoparticles, conventional NIR-emitting UCNPs known for their efficient UC (980-to-800 nm)^36,37^. TEM image shows that these UCNPs have a size of approximately 20.9 ± 1.5 nm, comparable to the WINPs used for comparison (Fig. S3). We further investigated the influence of Yb^3+^ concentration on PL efficiency by comparing the emission intensities with 10, 30, and 48% Yb^3+^ doping in both water and cyclohexane conditions. As a result, nanoparticles with lower Yb³⁺ content exhibited reduced quenching effects in water. However, for practical applications, we selected 48% Yb^3+^ doping for UCNPs as it provides an optimal balance between high absolute PL and quenching, making it well-suited for further applications (Fig. S4). Quantum yields (Q.Y.) of DS and UC were calculated to be 22.1 ± 0.9% and 3.1 ± 0.3%, respectively, as shown in Fig. S5. Moreover, compared to other lanthanide ions with similar down-shifting mechanisms, Nd-based nanomaterials show superior absorbance and quantum yield in NIR-I region (Table S1). In addition, to minimize the influence of shell thickness, we engineered shell structures with similar thicknesses, averaging 1.55 nm for WINPs and 1.35 nm for UCNPs (Fig. S6).

### Single-particle-level spectroscopy for evaluating photon population mechanism in each nanoparticle

**Fig. 2:**
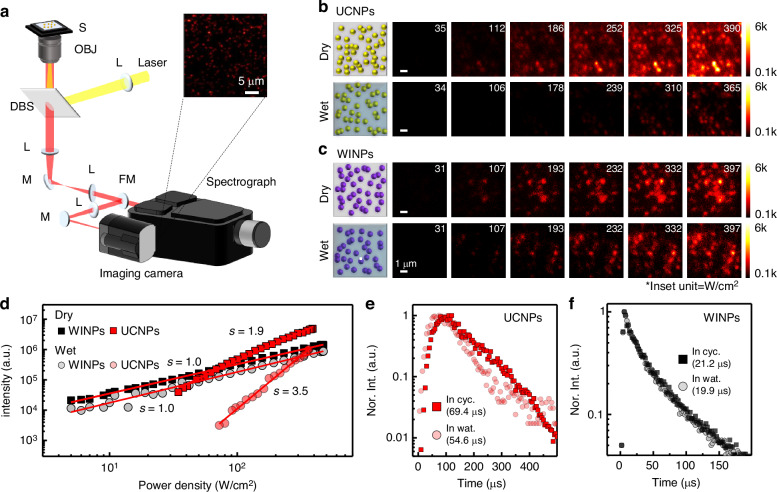
Characterization of the lifetime properties of WINPs. **a** Schematic of the microscopy setup for single-particle-level analysis. Imaging camera (using the flipping mirror) or spectrograph (without using the flipping mirror). S stage, L lens, DBS dichroic beamsplitter, OBJ objective lens, FM flipping mirror. Scale bar, 5 μm. **b** Single-level photoluminescence images of UCNPs under 980 nm excitation (power density of 0–10^3 ^W cm^−2^) in dry (top) and wet (bottom) conditions. **c** Single-level photoluminescence images of WINPs under 800 nm excitation (power density of 0–10^3 ^W cm^−2^) in dry (top) and wet (bottom) conditions. **d** Corresponding excitation-power-dependent photoluminescence emission intensity curves of WINPs and UCNPs. Slope *s*, indicates the slope in the log-log plot. **e** Photoluminescence (PL) lifetime decay profiles at 800 nm of UCNPs under 980 nm excitation, measured in cyclohexane and water, respectively. **f** PL lifetime decay profiles at 865 nm of WINPs under 800 nm excitation, measured in cyclohexane and water, respectively. (a.u. arbitrary units.)

To evaluate the photon population mechanism in WINPs and UCNPs, we conducted power-dependent PL measurements using a homemade wide-field spectroscopic imaging system (Fig. 2a) across a broad irradiance range of 5–10^3 ^W cm^−2^. The as-synthesized nanoparticles, initially coated with oleic acid and soluble in organic solvents, such as cyclohexane, underwent ligand exchange with dopamine hydrochloride via an ionization process to assess their performance in aqueous environments. Fourier transform infrared (FT–IR) spectra and zeta-potential measurements confirmed the successful replacement of the oleic acid ligand with an amine ligand (Fig. S7)^38,39^. As shown in Fig. 2a, the dopamine-coated nanoparticles were spin-coated onto a piranha-etched cover glass to obtain single-particle-level images.

Figure 2b displays images of the PL signal from the UCNPs at 800 nm under 980 nm irradiation, revealing a detection limit power density of 35 W cm^−2^ under dry conditions. In water, the detection limit power required to obtain the PL signal is approximately twice as high, i.e., 72 W cm^−2^. The corresponding NIR spectra and power-dependence curves are shown in Figs. S8 and 2d, respectively. To explore the effects of environmental changes on the dynamics in UCNPs, we analyzed the variations in their lifetime profiles, showing a decrease from 69.4 µs under dry conditions to 54.6 µs in water, a reduction of 21.3% (Fig. 2e). This change is attributed to the overtone transition of the O–H (ν = 3) at ~10,300 cm^−1^, closely matching the transition in Yb^3+^ ions (^2^F_5/2_ → ^2^F_7/2_) at ~10,200 cm^−1^ (Fig. S9). This spectral overlap resulted in a reduced lifetime and increased detection limit power owing to the enhanced quenching process, leading to a 10-fold overall reduction in the PL signals in water at the same power densities (Fig. 2d).

In contrast, the WINPs exhibited consistent PL signals at 865 nm under 800 nm irradiance with an identical detection limit power density of 5 W cm^−2^ in both dry and water environments (Fig. 2c). The negligible changes in the lifetime profiles (Fig. 2f) suggest that the energy transition in WINPs occurs independently of the water environment, significantly reducing signal quenching compared to that in UCNPs, with only a 1.8-fold reduction in signal intensity. To compare these quenching mechanisms, the relative population model illustrates (Fig. S10) that, unlike WINPs, energy quenching in the Yb^3+^/Tm^3+^ system of UCNPs is primarily attributed to nonradiative relaxation from Yb^3+^ to water molecules. These findings indicate that WINPs operate through a water-insensitive NIR-I-to-NIR-I DS process, requiring an approximately 15-fold lower power density because UC involves a complex multistep energy-absorption process, whereas DS is a simpler single-photon absorption process, leading to fewer energy losses during transfer. A lower-excitation source is particularly beneficial for biological environments because it effectively reduces background noise, enables highly sensitive signaling, and minimizes fluctuations by mitigating unwanted heating effects.

### Characterizations of incident light and evaluating nanoparticle performance in water environments

**Fig. 3:**
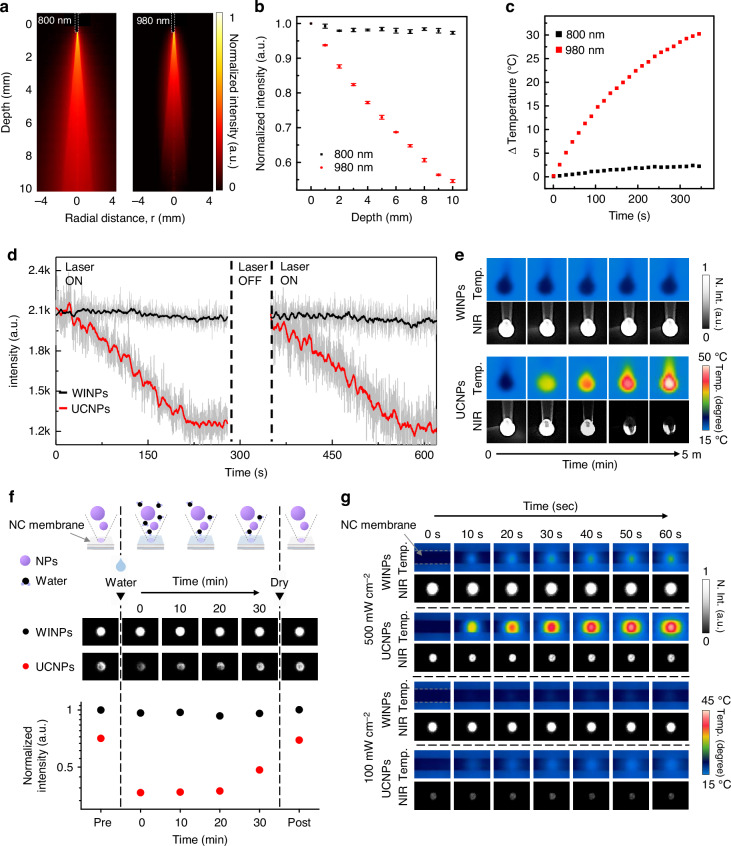
Characterization of incident lights. **a** Heatmap profiles for 800 and 980 nm light originating from the fiber (100 µm, NA = 0.22, *g* = 0.9, output power = 100 mW) placed in water solution, obtained via Monte Carlo simulation as a function of distance. **b** Measured intensity profiles of the two lasers as function of water solution depth (power density of 100 mW cm^−2^). **c** Temperature profiles over 5 min in a water solution, depending on the laser type, under irradiation at a power density of 2 W cm^−2^. **d** Time-dependent intensity profiles of two types of nanoparticles over 5 min. **e** Corresponding thermal and NIR intensity images of two types of nanoparticles. **f** NIR intensity images obtained from nitrocellulose (NC) membranes drop-cast with two types of nanoparticles (200 μg mL^−1^), then wetted with water. The membranes were stored in a desiccator, and the images were captured at 10-min intervals with a power density of 100 mW cm^−2^. The images labeled “Post” were captured after complete removal of the water in a vacuum oven. **g** Time-dependent thermal and NIR intensity images obtained from NC membranes drop-cast with two types of nanoparticles (200 μg mL^−1^), then wetted with water and irradiated at power densities of 500 and 100 mW cm^−2^ for 60 s. (a.u. arbitrary units)

Before applying them in practical biological applications, we examined the behavior of two types of incident light sources, 800 and 980 nm, i.e., the excitation wavelengths of WINPs and UCNPs, respectively, in aqueous environments. Using Monte Carlo simulations, we numerically analyzed the physics of light propagation in water, obtaining quantitative insights into the light distribution and penetration properties^40^. As shown in Fig. 3a, the simulation result for 100 mW photon packets indicated that photons with a wavelength of 800 nm propagated through more than 95% of the 10 mm thick water medium, whereas over 45% of photons at 980 nm were absorbed by water molecules. This phenomenon is attributed to the overtone of the O–H stretching mode of H_2_O (*ν* = 3, *ε*_980_ = 0.48 cm^−1^), which absorbs significantly more than in the NIR region at 800 nm (*ε*_800_ = 0.02 cm^−1^, Fig. S9).

The practical experimental results, depicted in Fig. 3b, demonstrate that at a wavelength of 800 nm, more than 97.3% of photons penetrated to a depth of 10 mm. In contrast, at a wavelength of 980 nm, 54.6% of the photons were absorbed within the same thickness, consistent with the simulation results. Considering these findings, achieving above 90% irradiance at a depth of 10 mm underwater would theoretically require significantly higher power output, ranging from 1 to 2 W, depending on the laser target area (Fig. S11). These power levels, especially at 980 nm, tend to produce unwanted thermal effects in water. When irradiating water with two types of lasers at the same power density (2 W cm^−2^), the 980 nm laser source resulted in a temperature increase of up to 50 degrees within 5 min, whereas the 800 nm laser source showed negligible thermal effect (Fig. 3c).

To investigate the correlation between the thermal effects and the performance of each nanoparticle, we observed time-dependent fluctuations in the intensities of the NIR-I signal under continuous laser irradiation for 5 min. As shown in Fig. 3d, the intensity fluctuation from WINPs at an 865 nm wavelength remained stable within 4% under 800 nm irradiation, as the water temperature did not increase. In contrast, intensities from UCNPs at an 800 nm wavelength decreased by up to 60% as the water temperature rose to 50 degrees within 5 min. Their corresponding thermal and NIR signal images are shown in Fig. 3e, and the NIR-I emission spectra are shown in Fig. S12. The stability of the laser was confirmed through power meter measurements, showing a fluctuation of less than 1.7% over a 1 h (Fig. S13). The signal degradation observed in UCNPs was attributed to the thermally induced emission quenching, where elevated temperatures enhanced the nonradiative relaxation pathways, leading to a significant decrease in the PL efficiency^41–44^. Although the signal attenuation from UCNPs can recover as the temperature drops, it is not suitable for applications involving small sample volumes or those requiring long-term measurements. In addition, a biomimetic environment was established using 2% intralipid phantoms to assess the NIR-I imaging capability. As shown in Fig. S14, WINPs-filled capillaries embedded in phantoms of varying thickness (~8 mm) exhibited enhanced resolution and 4-fold improvement in the S/B value under only irradiation at only 100 mW cm^−2^. These findings indicate that 800 nm irradiation of the WINPs ensures sustained high-quality signal output, allowing effective performance at low laser power in water-rich biological environments.

### Evaluation of WINPs for stable detection of target viruses under low power irradiation

To further explore the potential for biological application, we incorporated WINPs as nanoprobes into an LFA for detecting AIVs to validate their water-independent and highly stable detection capabilities. AIVs cause highly contagious diseases in poultry, leading to significant economic losses and posing a public health risk owing to their ability to infect humans^45–49^. Rapid and reliable detection of AIV is essential for effective prevention and control^50–52^. AIV detection is typically performed using opaque avian stools and cloacal swab samples. However, conventional LFAs using colorimetric or fluorescent signals in the ultraviolet or visible range often suffer from strong light scattering and absorption in such opaque samples^53,54^. These background interferences reduce signal clarity and detection reliability. In contrast, WINPs-LFA generates a background-free NIR signal, minimizing interference while maintaining high sensitivity for reliable AIV detection. To evaluate its diagnostic performance, the nucleoprotein within AIVs, a highly conserved internal viral protein less prone to mutations, was selected as the detection target^21,55^.

To assess whether WINPs could enhance the accuracy of LFA, we compared their performance to that of UCNPs on a nitrocellulose (NC) membrane, a key component of LFA strips. After adding 100 μL of water—typical LFA conditions—the WINPs demonstrated higher signal intensity and stability, with only a 4% fluctuation, whereas UCNPs exhibited a 40% signal degradation (Fig. 3f). Even after 30 min in a desiccator, the signal intensity of the UCNPs did not fully recover, indicating their limitations for water-based LFA applications. Full recovery occurred only after the removal of water in a vacuum oven, confirming that water-induced energy loss was responsible for degradation. As shown in Fig. 3g, irradiation of the NC membrane with a 980 nm laser at 500 mW cm^−2^ increased the water temperature to 43 degrees within 1 min, resulting in a 40% decrease in the PL signal of UCNPs. In contrast, the PL signal of WINPs remained stable within 6% fluctuation, as 800 nm irradiation caused negligible heating issues. Under 100 mW cm^−2^, thermal effects were minimal for both wavelengths; however, UCNPs exhibited significantly lower PL intensity. Consequently, WINPs, which demonstrated both higher and stable PL signal under low power conditions, are promising for biomarker detection in practical LFA applications.

**Fig. 4:**
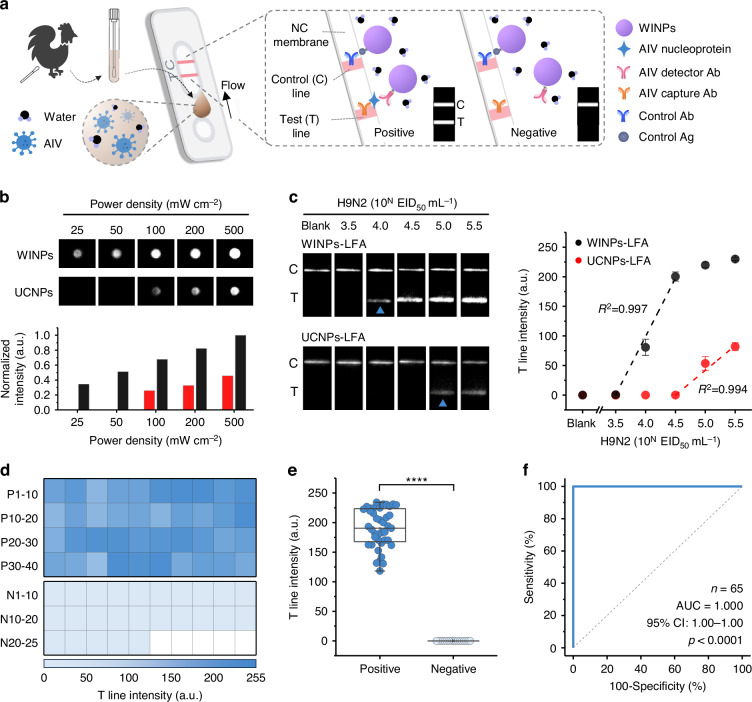
Evaluating WINP for stable detection of target viruses. **a** Schematic illustration of WINPs-LFA for detection of avian influenza viruses (AIVs). Ab antibody, Ag antigen. **b** NIR intensity images obtained from nitrocellulose (NC) membranes drop-cast with WINPs and UCNPs (200 μg mL^−1^) under different power densities of laser irradiation. **c** Responses of the WINPs-LFA and UCNPs-LFA to H9N2 AIVs at different concentration and standard curve of the test line (T line) intensities under low-power (100 mW cm^−2^) laser irradiation. **d** Heatmap of WINPs-LFA results using 65 clinical samples (40 AIV-positive and 25 AIV-negative). **e** Box and whisker plot of the WINPs-LFA for discriminating the AIV-positive samples from the AIV-negative samples. Boxes represent the mean, 25th and 75th percentiles, while whiskers show the largest and smallest values. **f** ROC curve and AUC analysis for AIV clinical samples. The AUC of 1 indicated high accuracy of the WINPs-LFA for AIV detection in clinical samples. AUC area under curve, CI confidence intervals. The test line intensities were obtained from the 8-bit ImageJ-processed images of LFA strips. Data are presented as mean (*n* = 3) values ± SD. Statistical analysis using a two-tailed unpaired *t* test: ****p < 0.0001

The fabrication of the WINPs-LFA for detecting AIVs involved coupling the WINPs with an anti-AIV nucleoprotein detector antibody (Ab) for the test line (T line) signal and a control antigen (Ag) for the control line (C line) signal through a series of modifications: amine-, maleimide-, and Ab-modification (Fig. S15 and “Methods”). AIV detection using the WINPs-LFA begins by loading an AIV sample solution onto the sample pad of the LFA strip (Fig. 4a). The target analyte, a nucleoprotein lysed from AIV, specifically binds to the detector Ab-WINPs, forming analyte-WINP conjugates. These conjugates flowed through the NC membrane, where AIV Abs on the T line capture them, resulting in an NIR signal. In contrast, in the absence of AIVs, an NIR signal was observed only on the C line because the conjugates were not captured on the T line. For the control experiments, UCNPs were applied using an identical procedure and denoted as UCNPs-LFA. NIR signals of both WINPs-LFA and UCNPs-LFA were captured under 100 mW cm^−2^ irradiation, the minimum power density required to compare their performances (Fig. 4b).

In this study, we assessed the analytical performance of the WINPs-LFA avian influenza H9N2 at concentrations ranging from 10^3.5^ to 10^5.5^ 50% egg infectious dose (EID_50_) mL^−1^. H9N2 is classified as a low-pathogenic virus but is recognized as a public health risk because of its reported ability to infect humans^56,57^. The T line intensities exhibited a linear relationship with increasing AIV concentrations, ranging from 10^3.5^ to 10^4.5^ EID_50_ mL^−1^, with a high correlation coefficient (*R*^2^) of 0.997 (Fig. 4c). Notably, the WINPs-LFA detected AIV concentrations as low as 10^4.0^ EID_50_ mL^−1^ (indicated by a triangle), whereas the UCNPs-LFA required a 10-fold higher concentration to produce a distinguishable T line intensity. The limit of detection (LOD) of the WINPs-LFA calculated using the IUPAC guidelines was 10^3.52^ EID_50_ mL^−1^, 10-fold lower compared to that of the UCNPs-LFA (10^4.53^ EID_50_ mL^−1^). While previous studies have reported a higher sensitivity of the NaYF_4_:Yb,Tm UCNPs-based LFA under high power conditions (Table S2), the WINPs-LFA demonstrated robust performance with significantly lower LODs, even under more practical, low power settings. These results can be attributed to the stable luminescence intensity of the WINPs, which maintains their performance under low power laser irradiation without energy loss, in contrast to the UCNPs that experience significant luminescence intensity reduction due to energy loss induced by water molecules.

To evaluate specificity, WINPs-LFA was tested using 10^5.0^ EID_50_ mL^−1^ of non-target avian pathogens, including infectious bronchitis virus (IBV), *Mycoplasma gallisepticum* (MG), *Mycoplasma synoviae* (MS), and Newcastle disease virus (NDV). The assay showed a significantly higher T line intensity for AIV than for the other pathogens (*p* < 0.0001), indicating the high specificity of WINPs-LFA with no detectable cross-reactivity (Fig. S16). Furthermore, the repeatability of the WINPs-LFA was evaluated by measuring coefficients of variation (CVs) for 10^4.0^ and 10^5.0^ EID_50_ mL^−1^ H9N2 AIVs. The intra-assay CVs were 1.14 and 4.52% and the inter-assay CVs were 2.54 and 5.28%, indicating the high repeatability of the WINPs-LFA (Fig. S17).

Clinical specimens such as avian cloacal swabs and blood are typically opaque and exhibit strong absorption in the visible range and negligible absorption in the NIR region. Consequently, the WINPs dispersed in these samples demonstrated stable NIR emission intensities without being disturbed by the background noise of the opaque sample matrix (Fig. S18a, b). We investigated the anti-interference capability of WINPs-LFA in detecting 10^4.0^ and 10^5.0^ EID_50_ mL^−1^ H9N2 AIVs in opaque avian cloacal swab samples and colored samples containing hemoglobin and bilirubin, respectively (Fig. S18c, d). The T line intensities of the WINPs-LFA showed no significant changes, even for AIVs spiked in these opaque solutions, demonstrating that WINPs-LFA is a reliable diagnostic tool for detecting target analytes in opaque clinical samples. Finally, we validated the clinical applicability of the WINPs-LFA in detecting AIVs under low power excitation conditions. We used 25 AIV-negative samples (cloacal samples) and 40 AIV-positive samples (oropharyngeal and vent swab samples) with different cycle threshold (Ct) values (Table S3). Figure 4d summarizes the T line intensities for each clinical sample, demonstrating significantly higher T line intensities for AIV-positive samples than for AIV-negative samples. The WINPs-LFA also yielded positive results for all AIV-positive samples without any false-negative results (*p* value < 0.0001) (Fig. 4e). We conducted a receiver operating characteristic (ROC) analysis, wherein the WINPs-LFA accurately distinguished AIVs in clinical samples with an area under the curve (AUC) of 1.000, indicating a sensitivity and specificity of 100% (Fig. 4f). Additionally, we compared the AIV detection results using WINPs-LFA and real-time PCR and observed a strong correlation between the two methods, as indicated by a Pearson’s coefficient (*r*) of −0.830 (Fig. S19).

## Discussion

In this study, we developed water-insensitive down-shifting nanoparticle (WINPs, NaYF_4_:5%Nd@NaYF_4_) that operate within the NIR-I range (700–900 nm) and demonstrated their potential as bio-suitable nanoprobes especially in water-rich environments. The optimized WINPs achieved a remarkable Q.Y. of 22.1 ± 0.9%, with an operational detection limit power 15-times lower than that of conventional UCNPs. These features support efficient utilization of the widely accessible 808 nm laser alongside silicon-based detectors, achieving lower background noise and enhanced performance even at low power density. Notably, single-particle-level spectroscopy confirmed that the WINPs maintained operational detection limit power under both dry and water conditions, offering consistent emission profiles regardless of the medium. Monte Carlo simulations and experimental results further demonstrated that 800 nm excitation minimizes thermal effects in water, making it more suitable for biological systems compared to the 980 nm excitation used in UCNPs. In water condition, WINPs exhibited only minimal fluctuations in PL intensity (<4%), underscoring their stability against laser-induced thermal quenching and their suitability for extended use in bio-environments. Furthermore, the result of NIR-I imaging in 2% intralipid phantoms demonstrated a clear improvement in resolution and signal-to-background ratio (S/B) under irradiation of 100 mW cm^−2^. Integrating WINPs into an LFA for detecting AIVs in opaque samples resulted in a 10-fold increase in sensitivity under a lower power irradiation of 100 mW cm^−2^. Conventional UCNP-based LFAs in previous studies typically required lasers with 10-fold higher power (1000 mW cm^−2^)^51,58^, which could rapidly increase temperature (>45 degrees) on the LFA strip within 1 min. This heating issue could denature proteins and disrupt immunoreactions, compromising the assay accuracy. In contrast, low power irradiation caused negligible thermal effects on LFA strip. Therefore, WINPs-LFA, which maintains high and stable signal under low power irradiation, enables reliable and accurate biosensing. Moreover, as summarized in Table S4, other NIR-based LFAs have required high-power excitation sources (often up to several watts) or expensive InGaAs detectors with complex cooling systems, limiting their practical applicability. In contrast, WINPs-LFA exhibited high sensitivity with low-power excitation and a cost-effective silicon-based detector, making it a more practical alternative for biosensing. This system exhibited excellent specificity and repeatability, with 100% sensitivity confirmed through ROC analysis. Overall, these results highlighted the potential of WINPs to replace conventional NIR-emitting phosphors, particularly in applications requiring low-power excitation and water-rich biological environments.

## Materials and methods

### Reagents

Yttrium(III) acetate hydrate (99.9%), neodymium(III) acetate hydrate (99.9%), ytterbium(III) acetate hydrate (99.9%), thulium(III) acetate hydrate (99.95%), oleic acid (OA, 90%), 1-octadecene (ODE, 90%), sodiumhydroxide (NaOH, ≥98%), ammoniumfluoride (NH4F, ≥99.9%), ethanol (absolute), methanol (≥99.8%), cyclohexane (≥99%), tetra-hydrofuran (THF, ≥99.9%), dopamine hydrochloride (≥99.9%), hydro-chloric acid (HCl, 37%), dimethyl sulfoxide (DMSO, ≥99.9%), hydroxylamine hydrochloride (HH, 99%), bovine serum albumin (BSA), Tween 20, sucrose, polyvinylpyrrolidone (PVP, 10k) and *N*,*N*-dimethylformamide anhydrous (DMF, ≥99.8%) were purchased from Sigma-Aldrich, St. Louis, MO, USA. HEPES buffer (1 M) was purchased from Gibco. *N*-succinimidyl *S*-acetylthioacetate (SATA), and sulfosuccinimidyl 4-(*N*-maleimidomethyl) cyclohexane-1-carboxylate (sulfo-SMCC) were purchased from Thermo Fisher Scientific, Waltham, MA, USA. An Amicon Ultra centrifugal filter (0.5 mL, 30 K, 5 K) was purchased from Millipore, Bedford, MA, USA. The NC membrane strips, sample pads, conjugated pads, absorbent pads, anti-AIV detector antibody, malaria antigen, and the inactivated AIV antigens were provided by Median Diagnostic (Republic of Korea).

### Synthesis of lanthanide-doped nanoparticles

Lanthanide-doped nanoparticles were synthesized via thermal decomposition of lanthanide acetate precursors. In a typical synthesis, Ln(CH_3_CO_2_)_3_ (Ln = Y and Nd: 0.5–90%, total 0.4 mmol) or (Ln = Y, Yb, and Tm total 0.4 mmol) was combined with 3 mL of oleic acid and 7 mL of 1-octadecene. This mixture was heated to 150 °C for 60 min, then cooled to 50 °C. Subsequently, a methanolic solution of NaOH (1 mmol, 2 mL) and NH_4_F (1.6 mmol, 4 mL) was gradually added to the oleate-lanthanide solution while stirring at 50 °C. The solution was then heated to 100 °C and degassed using vacuum pump to eliminate residual water and methanol. Next, the solution was heated to 300 °C and maintained under argon for 1 h before cooling to room temperature. The precipitated nanoparticles were collected by centrifugation, washed, and finally re-dispersed in 2 mL of cyclohexane. Core@shell nanoparticles were synthesized following a procedure similar to that used to synthesize core nanoparticle.

### Estimation of critical distance (*R*_c_) between Nd^3+^ ions

According to the literature, *R*_c_ between Nd^3+^ ions could be derived by Blasse’s equation:

$${R}_{{\rm{c}}}=2\left({\frac{3V}{4{\rm{\pi }}{x}_{c}N}}\right)^{1/3}$$

where *x*_*c*_ denotes the concentrations of Nd^3+^ ion, *N* represents the number of lattice sites within the unit cell that can be occupied by emitter ions, *V* is the volume of the unit cell. In the case of hexagonal NaYF_4_ (JCPDS 28-1192) with a space group of *P6*_*3*_/*m* (*N* = 1.5), the unit cell parameters are *α* = 5.96 Å and *c* = 3.53 Å, resulting in a unit cell volume of 108.6 Å^3^. Consequently, for a 5% doping level, *R*_c_ was estimated to be about 14.0 Å (Fig. S2).

### Quantum yield measurement

Quantum yields (Q.Y.) of WINP (NaYF_4_:5%Nd@NaYF_4_) and UCNP (NaYF_4_:48%Yb,2%Tm@NaYF_4_) measured using the integrating sphere connected to a fiber optic spectrometer (Fig. S5). Initially, the setup was calibrated using a Xenon Arc light sources (THORLABS) to derive the correction factor, which was subsequently used to adjust the measured spectra of the sample. Then, micro quartz cuvette containing a cyclohexane solution of the nanoparticles was positioned inside the integrating sphere, connected to the fiber optics spectrometer (Flame, Ocean Optics), to capture the emission and excitation light signals under 980 and 800 nm excitation. A cyclohexane solution without nanoparticles was used as a reference. The Q.Y. was then calculated using the following equation:

$${\rm{Q}}.{\rm{Y}}.={N}_{{{\rm{em}}}}/{N}_{{{\rm{abs}}}}=\int {P}_{{{\rm{em}}}}(\lambda )\lambda d\lambda \,/\int [{P}_{{{\rm{ref}}}}(\lambda )-{P}_{{{\rm{sample}}}}(\lambda )]\lambda d\lambda$$

where ${P}_{{{\rm{em}}}}(\lambda )$ is the emission intensity spectrum of the sample, ${P}_{{{\rm{ref}}}}(\lambda )$ and ${P}_{{{\rm{sample}}}}(\lambda )$ are the intensity spectra of excitation light recorded after passing through the reference sample, respectively.

### Single-particle-level wide-field imaging and spectroscopy

Two types of nanoparticles were imaged using a homemade wide-field spectroscopic imaging system^59^. Before surface modification, the nanoparticles were dispersed in hexane, and those modified with dopamine were dispersed in water. Samples were prepared by spin-coating 10 μL of each nanoparticle dispersed in hexane or water onto piranha-etched cover glass. The dry samples were imaged as spin-coated, while for wet samples, 20 μL of water were dropped onto the spin-coated nanoparticles before imaging. The samples on the cover glass were placed on an oil immersion-type objective lens (UPlanSApo 60X, Olympus) in an inverted microscope (IX71, Olympus). For the excitation of each nanoparticle, a 795 nm laser from a continuous-wave laser diode (QFLD-795-100S, QPhotonics) mounted on a laser diode driver (MW-SN-0905-0029, MW Technologies) and a 980 nm laser from a CW laser diode (AC1401-0600-0980-SM, Gooch & Housego) mounted on a laser diode driver (CLD1015, Thorlabs) were used, respectively. The 795 and 980 nm laser beams were collimated using lens (LA-1027-B-ML, Thorlabs and LA1509-B-ML, Thorlabs, respectively). The nanoparticles on the samples were excited by wide-field illumination using another lens (AC508-300-B-ML, Thorlabs). Dichroic beamsplitters (Di02-830R, Semrock for WINP and ZT1064rdc-sp, Chroma for conventional NIR nanoparticle) and optical filters (LP02-808RE-25, Semrock for WINP and FF01-890/SP-25, Semrock for conventional NIR nanoparticle) were used to block the excitation wavelength and gather the photoluminescence emitted from the nanoparticles. Emission images were collected using an electron-multiplying charge-coupled device (EMCCD; iXon3 888, DU-888E-C00-#BV, Andor). Spectroscopic measurements were performed using a CCD camera (PIXIS 400 BR, Princeton Instruments) mounted on a spectrograph (IsoPlane SCT 320, Princeton Instruments).

### Monte Carlo simulation for light propagation

We conducted Monte Carlo simulation in MATLAB, employing a technique similar to that previously reported^40^. Photon packet, each with an initial energy *E*_0_, were delivered into the water, assuming an angular distribution from an optical fiber with a numerical aperture NA = 0.22 and anisotropy parameter *g* = 0.9. In non-scattering media such as water, the absorption coefficients were determined to be *ε*_980_ = 4 × 10^−3^ cm^−1^ M^−1^, *ε*_800_ = 0.1, respectively. The index of refraction of water is calculated to be *n* = 1.33. The photon packets were uniformly distributed across a 100 μm region in the center of the simulation domain, representing a 100 μm diameter optical fiber. The simulation domain, measuring 10 mm in depth and 15 mm in width, was divided into bins.

### Dopamine-modified nanoparticles

Nanoparticles (15 mg) were dissolved in THF, while dopamine hydrochloride (50 mg) was dissolved in water. These solutions were combined in a 50 mL flask and heated to 50 °C with vigorous stirring. After adding hydrochloric acid (HCl), the resulting NH_2_-ligand was collected through several washing steps.

### Ligand exchange with antibody-modified nanoparticles

NH_2_-modified nanoparticles (2 mg) were dispersed in 200 µL of HEPES buffer (10 mM). Sulfo-SMCC was also dispersed in HEPES buffer (10 mM), and the two solutions were mixed and incubated for 5 h. A SATA stock solution was added to the pristine AIV-detector antibody (150 µg) and incubated for 30 min. Subsequently, a hydroxylamine hydrochloride (HH) solution (1.75 µL, 0.5 M) was added and incubated for 2 h. The resulting solution was washed using a 30 kDa centrifuge filter. Thiol-modified antibodies and maleimide-modified NPs were then linked through a click reaction. Finally, the surface of the AIV detector antibody-NPs was blocked with a 1% BSA solution to minimize non-specific binding. The Ab-immobilized WINPs (Ab-WINPs) were characterized by zeta potential measurements, FT-IR, and ultraviolet-visible (UV-vis) absorbance analysis. The zeta potential of the WINPs decreased from +35.0 mV to −36.1 mV after maleimide modification, which then increased to −15.2 mV after Ab immobilization (Fig. S14b). FT-IR analysis showed new peaks at 1542 and 1639 cm^−1^ (amide I and amide II)^60,61^ appeared after Ab immobilization (Fig. S14c). Additionally, the Ab-WINPs showed absorption peak in the 280 nm (Fig. S14d), successful antibody conjugation through a thiol-maleimide reaction between the maleimide groups on the WINPs and the thiol groups on the Abs^58^.

### Fabrication of the WINPs-LFA strip

A NC membrane, dispensed with a 1.0 mg mL^−1^ of anti-AIV capture antibody and 1.0 mg mL^−1^ of anti-malaria antibody in the test and control line, respectively, was provided by Median Diagnostic (Republic of Korea). A conjugate pad was pretreated with 15 μL of blocking reagents [1.5% (w/v) BSA, 2% (w/v) sucrose, 1.5% (w/v) Tween 20, and 0.25% (w/v) PVP in water]. After drying at 30 °C for 30 min in a vacuum oven, anti-AIV detector antibody-conjugated WINPs and malaria antigen-conjugated WINPs were dried on the conjugate pad at 30 °C for 30 min in a vacuum oven. Finally, the LFA strip was fabricated by stacking the as-prepared NC membrane, as-pretreated conjugate pad, sample pad, and absorbent pad with a 2 mm overlap on an adhesive backing card and cut to a width of 4 mm. A conjugate pad, sample pad, absorbent pad, and backing card were provided by Median Diagnostic (Republic of Korea).

### AIV detection using the WINPs-LFA

We evaluated analytical sensitivity of the WINPs-LFA using different concentrations of AIV (0, 2 × 10^3.5^, 2 × 10^4.0^, 2 × 10^4.5^, 2 × 10^5.0^, 2 × 10^5.5^ EID_50_ mL^−1^), which were prepared by serial dilution using PBS buffer (pH 7.4). The AIV samples were mixed at a ratio of 1:1 with the loading buffer, which facilitates the lysis of the nucleoprotein (NP) within AIV. The NIR luminescence signal of WINPs was measured 15 min after adding 100 μL of AIV samples to the sample pad using an intensified sCMOS camera with an 850 nm longpass filter (Thorlabs, FELH0850), under excitation at 800 nm with an 800 nm band pass filter (Semrock, ff-01-800/12-25). An additional 75 mm focal length lens (Edmund, #54-691) was fixed at the C-mount thread of the camera. For the control experiments, UCNPs were also applied in an identical procedure, denoted as UCNPs-LFA. The NIR luminescence signal of UCNPs were measured using an intensified sCMOS camera with an 800 nm band pass filter, under excitation at 980 nm. The AIV samples and loading buffer were provided by Median Diagnostic (Republic of Korea).

We validated the clinical applicability of the WINPs-LFA using 65 clinical samples including 40 AIV positive and 25 AIV negative samples. The positive samples, collected at 1dpc (days post challenge), 3dpc, and 5dpc, were provided by Konkuk University and Kyungpook National University. The animal experiment procedures were approved by the institutional animal care and use committee of Korea University College of Medicine (Protocol Number: KUIACUC-2023-246) and performed in accordance with the regulations and guidelines of this committee. The negative samples were collected as cloacal swab of uninfected avians and provided by Median Diagnostic (Republic of Korea). The samples were confirmed by real-time PCR. 100 μL of samples mixed at a ratio of 1:1 with the loading buffer were added to the sample pad. Then the AIV detection was performed as described above.

### Statistical analysis

Statistical analyses were performed using Origin Pro 2016 and IBM SPSS statistical package (version 27). Means and ±standard deviations were calculated for each data point from at least triplicate measurements. The LOD was calculated using the IUPAC guidelines [LOD = mean + 3.3*σ* of the blank; *σ* is standard deviation]. Statistical significance of differences between AIV positive samples and AIV negative samples was evaluated using a two-tailed unpaired *t*-test (*p < 0.05, **p < 0.01, ***p < 0.001, and ****p < 0.0001).

### Characterizations

Sample images were analyzed on a JEN-2100F (JEOL. Ltd.) installed at the Hanyang LINC3.0 Center for Research Facilities (Seoul, Republic of Korea). The XRD patterns of the NPs were recorded by an XRD-7000 diffractometer. A Zetasizer Nano ZSP instrument (Malvern Co., UK) was used to determine the zeta potentials of the WINPs. The Fourier transform infrared (FT-IR) spectra of the WINPs were obtained by using an iS50 Fourier transform infrared spectrophotometer (Thermo Fisher Scientific Co., USA) installed at Center for Polymers and Composite Materials (Hanyang University, Seoul, Republic of Korea). The photoluminescence emission spectra were recorded using a Flame spectrometer (Ocean Optics, Inc., USA) under external excitation at 800 or 980 nm provided by an infrared diode laser (Changchun New Industries Optoelectronics Tech. CO., China). A Jasco V-700 UV-Vis spectrophotometer (Jasco., Japan) was used to record the absorbance spectra. The lifetime and images were obtained by the iStar intensified sCMOS camera (Andor) using time-gated imaging technology. Their time gate delay was 100 μs, and the time gate width was 50 μs. All practical images were obtained under the irradiation of an 808 nm and 980 nm laser (CNI, MDL-H-808-5W, MDL-III-980-2W) with a power density of 0.5 W cm^−2^. The NIR emission wavelength was selectively measured using a bandpass filter (Semrock, ff-01-800/12-25, Edmund 980 nm CWL, 12.5 mm Dia., Hard Coated OD 4.0 10 nm), longpass filter (Thorlab, FELH0850) and shortpass filter (Semrock, ff-01-950/sp-25) placed in front of an all detectors. The optical properties were measured using a tunable laser EKSPLA NT-342.

## Supplementary information

Supplemental Materials

## Data Availability

The authors declare that all data supporting the finding of this study are available within the paper and its Supplementary Information files. The remaining data are available within the article, Supplementary Information, or Source data file.

## References

[1] Lamon, S. et al. Lanthanide ion-doped upconversion nanoparticles for low-energy super-resolution applications. *Light Sci. Appl.* **13**, 252 (2024). 39277593 10.1038/s41377-024-01547-6 PMC11401911

[2] Li, H. H., Wang, Y. K. & Liao, L. S. Near-Infrared luminescent materials incorporating rare earth/transition metal ions: from materials to applications. *Adv. Mater.* **36**, 2403076 (2024). 10.1002/adma.202403076 38733295

[3] Fan, Y. et al. Lifetime-engineered NIR-II nanoparticles unlock multiplexed in vivo imaging. *Nat. Nanotechnol.* **13**, 941–946 (2018). 30082923 10.1038/s41565-018-0221-0

[4] Pei, P. et al. X-ray-activated persistent luminescence nanomaterials for NIR-II imaging. *Nat. Nanotechnol.* **16**, 1011–1018 (2021). 34112994 10.1038/s41565-021-00922-3

[5] Liu, C. et al. Emerging advances in lanthanide photon avalanche nanophotonics. *Nano Lett.* **24**, 15489–15500 (2024). 39576321 10.1021/acs.nanolett.4c04524

[6] Wang, T. et al. A hybrid erbium(III)–bacteriochlorin near-infrared probe for multiplexed biomedical imaging. *Nat. Mater.* **20**, 1571–1578 (2021). 34326504 10.1038/s41563-021-01063-7

[7] Chen, Y., Wang, S. F. & Zhang, F. Near-infrared luminescence high-contrast in vivo biomedical imaging. *Nat. Rev. Bioeng.* **1**, 60–78 (2023).

[8] Hong, G. S., Antaris, A. L. & Dai, H. J. Near-infrared fluorophores for biomedical imaging. *Nat. Biomed. Eng.* **1**, 0010 (2017).

[9] Yang, Y. W. et al. Fluorescence-amplified nanocrystals in the second near-infrared window for in vivo real-time dynamic multiplexed imaging. *Nat. Nanotechnol.* **18**, 1195–1204 (2023). 37349506 10.1038/s41565-023-01422-2

[10] Lee, C. et al. Giant nonlinear optical responses from photon-avalanching nanoparticles. *Nature* **589**, 230–235 (2021). 33442042 10.1038/s41586-020-03092-9

[11] Lee, C. et al. Indefinite and bidirectional near-infrared nanocrystal photoswitching. *Nature* **618**, 951–958 (2023). 37258675 10.1038/s41586-023-06076-7

[12] Liang, L. L. et al. Energy flux manipulation in upconversion nanosystems. *Acc. Chem. Res.* **52**, 228–236 (2019). 30557000 10.1021/acs.accounts.8b00469

[13] Du, P. Y. et al. Near-infrared-responsive rare earth nanoparticles for optical imaging and wireless phototherapy. *Adv. Sci.* **11**, 2305308 (2024). 10.1002/advs.202305308 PMC10885668 37946706

[14] Li, H. et al. Lanthanide-doped near-infrared nanoparticles for biophotonics. *Adv. Mater.* **33**, 2000678 (2021). 10.1002/adma.202000678 32638426

[15] Du, K. M. et al. Nanocomposites based on lanthanide-doped upconversion nanoparticles: diverse designs and applications. *Light Sci. Appl.* **11**, 222 (2022). *volume*. 35831282 10.1038/s41377-022-00871-z PMC9279428

[16] Kang, D. et al. A local water molecular-heating strategy for near-infrared long-lifetime imaging-guided photothermal therapy of glioblastoma. *Nat. Commun.* **14**, 2755 (2023). 37179387 10.1038/s41467-023-38451-3 PMC10183012

[17] Wu, Y. K. et al. Lanthanide luminescence nanothermometer with working wavelength beyond 1500 nm for cerebrovascular temperature imaging in vivo. *Nat. Commun.* **15**, 2341 (2024). 38491065 10.1038/s41467-024-46727-5 PMC10943110

[18] Ming, J. et al. High-brightness transition metal-sensitized lanthanide near-infrared luminescent nanoparticles. *Nat. Photonics* **18**, 1254–1262 (2024). 10.1038/s41596-025-01245-6 40908364

[19] Dibaba, S. T. et al. Recent progress of energy transfer and luminescence intensity boosting mechanism in Nd^3+^-sensitized upconversion nanoparticles. *J. Rare Earths* **37**, 791–805 (2019).

[20] Nazir, J. et al. Persistence of avian influenza viruses in lake sediment, duck feces, and duck meat. *Appl. Environ. Microbiol.* **77**, 4981–4985 (2011). 21622783 10.1128/AEM.00415-11 PMC3147373

[21] Park, S. et al. Detection of avian influenza virus from cloacal swabs using a disposable well gate FET sensor. *Adv. Healthc. Mater.* **6**, 1700371 (2017). 10.1002/adhm.201700371 28509437

[22] Callison, S. A. et al. Development and evaluation of a real-time Taqman RT-PCR assay for the detection of infectious bronchitis virus from infected chickens. *J. Virol. Methods* **138**, 60–65 (2006). 16934878 10.1016/j.jviromet.2006.07.018 PMC7112890

[23] Liu, Q. et al. Upconversion luminescence imaging of cells and small animals. *Nat. Protoc.* **8**, 2033–2044 (2013). 24071909 10.1038/nprot.2013.114

[24] Würth, C. et al. Quantum yields, surface quenching, and passivation efficiency for ultrasmall core/shell upconverting nanoparticles. *J. Am. Chem. Soc.* **140**, 4922–4928 (2018). 29570283 10.1021/jacs.8b01458

[25] Li, F. et al. Size-dependent lanthanide energy transfer amplifies upconversion luminescence quantum yields. *Nat. Photonics* **18**, 440–449 (2024).

[26] Liang, L. L. et al. Continuous-wave near-infrared stimulated-emission depletion microscopy using downshifting lanthanide nanoparticles. *Nat. Nanotechnol.* **16**, 975–980 (2021). 34127821 10.1038/s41565-021-00927-y

[27] Loh, K. Y. et al. Sharp-peaked lanthanide nanocrystals for near-infrared photoacoustic multiplexed differential imaging. *Commun. Mater.* **5**, 164 (2024). 40322261 10.1038/s43246-024-00605-1 PMC12048011

[28] Kang, D. et al. Self-referenced dual-near-infrared emission-based sensor platform for the ultrasensitive discrimination of D_2_O and H_2_O. *Sens. Actuators B Chem.* **401**, 134948 (2024).

[29] Skripka, A. et al. Inert shell effect on the quantum yield of neodymium-doped near-infrared nanoparticles: the necessary shield in an aqueous dispersion. *Nano Lett.* **20**, 7648–7654 (2020). 32941042 10.1021/acs.nanolett.0c03187

[30] Huang, J. S. et al. Cross relaxation enables spatiotemporal color-switchable upconversion in a single sandwich nanoparticle for information security. *Adv. Mater.* **36**, 2310524 (2024). 10.1002/adma.202310524 38150659

[31] Stroud, J. S. Concentration quenching of Nd^3+^ fluorescence. *Appl. Opt.* **7**, 751–757 (1968). 20068677 10.1364/AO.7.000751

[32] Carneiro et al. Theoretical and experimental investigation of the Tb^3+^ → Eu^3+^ energy transfer mechanisms in cubic A_3_Tb_0.90_Eu_0.10_(PO_4_)_3_ (A = Sr, Ba) materials. *J. Phys. Chem. C* **124**, 10105–10116 (2020).

[33] Sontakke, A. D. et al. Unraveling the Eu^2+^ → Mn^2+^ energy transfer mechanism in w-LED phosphors. *J. Phys. Chem. C* **124**, 13902–13911 (2020).

[34] Blasse, G. Energy transfer in oxidic phosphors. *Phys. Lett. A* **28**, 444–445 (1968).

[35] Huang, K. et al. Room-temperature upconverted superfluorescence. *Nat. Photonics* **16**, 737–742 (2022).

[36] Baride, A. et al. A NIR-to-NIR upconversion luminescence system for security printing applications. *RSC Adv.* **5**, 101338–101346 (2015).

[37] Kang, D. et al. An efficient NIR-to-NIR signal-based LRET system for homogeneous competitive immunoassay. *Biosens. Bioelectron.* **150**, 111921 (2020). 31818754 10.1016/j.bios.2019.111921

[38] Li, X. J. et al. Enhanced corrosion and wear resistance via dopamine-functionalized Ti_3_C_2_T_x_ MXene/waterborne polyurethane coating on magnesium alloy. *Mater. Today Chem.* **39**, 102142 (2024).

[39] Hlaváček, A. et al. Bioconjugates of photon-upconversion nanoparticles for cancer biomarker detection and imaging. *Nat. Protoc.* **17**, 1028–1072 (2022). 35181766 10.1038/s41596-021-00670-7

[40] Stujenske, J., Spellman, T. & Gordon, J. Modeling the spatiotemporal dynamics of light and heat propagation for in vivo optogenetics. *Cell Rep.* **12**, 525–534 (2015). 26166563 10.1016/j.celrep.2015.06.036 PMC4512881

[41] Liu, X. L. et al. Fast wide-field upconversion luminescence lifetime thermometry enabled by single-shot compressed ultrahigh-speed imaging. *Nat. Commun.* **12**, 6401 (2021). 34737314 10.1038/s41467-021-26701-1 PMC8568918

[42] Yu, D. C., Ballato, J. & Riman, R. E. Temperature-dependence of multiphonon relaxation of rare-earth ions in solid-state hosts. *J. Phys. Chem. C* **120**, 9958–9964 (2016). 10.1021/acs.jpcc.6b01466 PMC5388458 28408967

[43] Dang, P. P. et al. How to obtain anti-thermal-quenching inorganic luminescent materials for light-emitting diode applications. *Adv. Opt. Mater.* **10**, 2102287 (2022).

[44] Wang, Y. Z., Chen, B. & Wang, F. Overcoming thermal quenching in upconversion nanoparticles. *Nanoscale* **13**, 3454–3462 (2021). 33565549 10.1039/d0nr08603g

[45] Kim, J. Y. et al. Detection of intercontinental reassortant H6 avian influenza viruses from wild birds in South Korea, 2015 and 2017. *Front. Vet. Sci.* **10**, 1157984 (2023). 37377949 10.3389/fvets.2023.1157984 PMC10291271

[46] Pinotti, F. et al. Modelling the transmission dynamics of H9N2 avian influenza viruses in a live bird market. *Nat. Commun.* **15**, 3494 (2024). 38693163 10.1038/s41467-024-47703-9 PMC11063141

[47] Caserta, L. C. et al. Spillover of highly pathogenic avian influenza H5N1 virus to dairy cattle. *Nature* **634**, 669–676 (2024). 39053575 10.1038/s41586-024-07849-4 PMC11485258

[48] Shi, W. F. & Gao, G. F. Emerging H5N8 avian influenza viruses. *Science* **372**, 784–786 (2021). 34016764 10.1126/science.abg6302

[49] Neumann, G. & Kawaoka, Y. Highly pathogenic H5N1 avian influenza virus outbreak in cattle: the knowns and unknowns. *Nat. Rev. Microbiol.* **22**, 525–526 (2024). 39060613 10.1038/s41579-024-01087-1 PMC12720498

[50] Kang, D. et al. An NIR dual-emitting/absorbing inorganic compact pair: a self-calibrating LRET system for homogeneous virus detection. *Biosens. Bioelectron.* **190**, 113369 (2021). 34098357 10.1016/j.bios.2021.113369

[51] Kim, J. et al. Rapid and background-free detection of avian influenza virus in opaque sample using NIR-to-NIR upconversion nanoparticle-based lateral flow immunoassay platform. *Biosens. Bioelectron.* **112**, 209–215 (2018). 29709831 10.1016/j.bios.2018.04.047

[52] Jung, H. et al. A size-selectively biomolecule-immobilized nanoprobe-based chemiluminescent lateral flow immunoassay for detection of avian-origin viruses. *Anal. Chem.* **93**, 792–800 (2021). 33175513 10.1021/acs.analchem.0c03153

[53] Song, Z. R. et al. Enhanced NIR-II fluorescent lateral flow biosensing platform based on supramolecular host-guest self-assembly for point-of-care testing of tumor biomarkers. *ACS Appl. Mater. Interfaces* **15**, 52038–52050 (2023). 10.1021/acsami.3c14339 37886790

[54] Liu, S. J. et al. Evaluation of the multidimensional enhanced lateral flow immunoassay in point-of-care nanosensors. *ACS Nano* **18**, 27167–27205 (2024). 39311085 10.1021/acsnano.4c06564

[55] Miyoshi-Akiyama, T. et al. Discrimination of influenza A subtype by antibodies recognizing host-specific amino acids in the viral nucleoprotein. *Influenza Other Respir. Viruses* **6**, 434–441 (2012). 22329815 10.1111/j.1750-2659.2012.00335.x PMC6505565

[56] Kariithi, H. M. et al. Genetic characterization and pathogenesis of the first H9N2 low pathogenic avian influenza viruses isolated from chickens in Kenyan live bird markets. *Infect. Genet. Evol.* **78**, 104074 (2020). 31634645 10.1016/j.meegid.2019.104074

[57] Pusch, E. A. & Suarez, D. L. The multifaceted zoonotic risk of H9N2 avian influenza. *Vet. Sci.* **5**, 82 (2018). 30248906 10.3390/vetsci5040082 PMC6313933

[58] Kim, S. et al. On-site remote monitoring system with NIR signal-based detection of infectious disease virus in opaque salivary samples. *ACS Sens.* **8**, 1299–1307 (2023). 36786758 10.1021/acssensors.2c02818

[59] Kim, J. et al. Universal emission characteristics of upconverting nanoparticles revealed by single-particle spectroscopy. *ACS Nano* **17**, 648–656 (2023). 36565305 10.1021/acsnano.2c09896

[60] Kim, K. et al. Metallic woodpile nanostructures for femtomolar sensing of Alzheimer’s neurofilament lights. *ACS Nano* **14**, 10376–10384 (2020). 32706577 10.1021/acsnano.0c04053

[61] Song, Y. J. et al. Oxygen-enriching triphase platform for reliable sensing of femtomolar Alzheimer’s neurofilament lights. *Biosens. Bioelectron.* **260**, 116431 (2024). 38815462 10.1016/j.bios.2024.116431

