# Supplementary material for: Water-insensitive NIR-I-to-NIR-I down-shifting nanoparticles enable stable biomarker detection at low power thresholds in opaque aqueous environments
Source: Light Sci Appl. 2025 Jul 3;14:235. doi: 10.1038/s41377-025-01882-2 (PMC12229501; doi:10.1038/s41377-025-01882-2)
Supplement: Supplementary file 1 — Supplemental Materials [file 41377_2025_1882_MOESM1_ESM.docx]

**Supplementary Information**

Water-insensitive NIR-I-to-NIR-I Down-shifting Nanoparticles Enable Stable Biomarker Detection at Low Power Thresholds in Opaque Aqueous Environments

Dongkyu Kang^1,^*, Suyeon Kim^1,^*, Yeongchang Goh^2^, Minseo Kim^2^, Sun-Hak Lee^3^, Jung-Hoon Kwon^4^, Sang Hwan Nam^2,^**, Joonseok Lee^1,5,^**

^1^Department of Chemistry, Hanyang University, Seoul 04763, Republic of Korea

^2^Laboratory of Nanoscopic imaging & Spectroscopy Analysis, Chemical Analysis Center, Korea Research Institute of Chemical Technology, Daejeon 34114, Republic of Korea

^3^Avian Disease Laboratory, College of Veterinary Medicine, Konkuk University, 120 Neungdong-ro, Gwangjin-gu, Seoul 143-701, Republic of Korea

^4^Laboratory of Veterinary Microbiology, College of Veterinary Medicine, Kyungpook National University, 80 Daehak-ro, Daegu 41566, Republic of Korea

^5^Research Institute for Convergence of Basic Sciences, Hanyang University, Seoul 04763, Republic of Korea

**These authors contributed equally*

***Corresponding author*

*Email address:* *joonseoklee@hanyang.ac.kr, shnam@krict.re.kr*


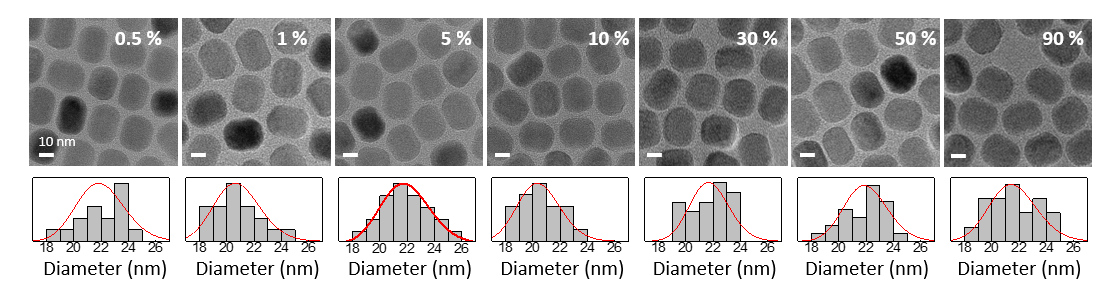


**Supplementary Figure 1.** Characterizations of series of NaYF_4_:x%Nd@NaYF_4_ (x mol%, x: 0.5 – 90%) nanoparticles. TEM images of nanoparticles (top), size distribution with 0.5%: 22.0 ± 1.7 nm, 1%: 20.8 ± 1.7 nm, 5%: 21.9 ± 1.8 nm, 10%: 20.4 ± 1.5 nm, 30%: 21.7 ± 1.4 nm, 50%: 22.0 ± 1.5 nm, and 90%: 21.6 ± 1.7 nm (bottom). Scale bars: 10 nm.


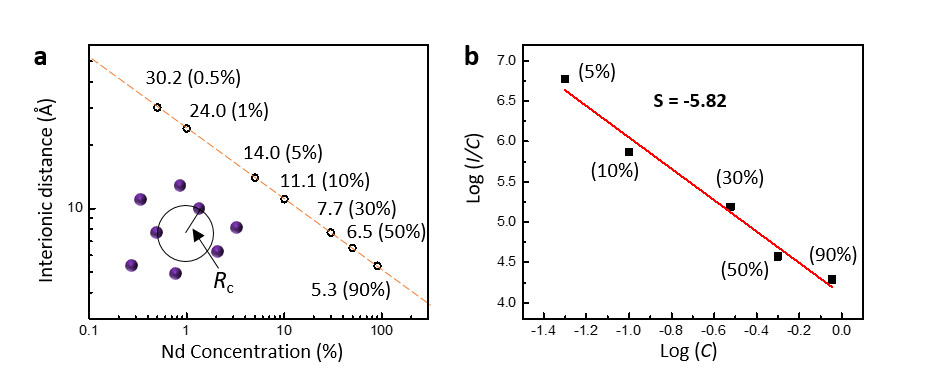


**Supplementary Figure 2.** Characterizations of series of NaYF_4_:x%Nd@NaYF_4_ (x mol%, x: 0.5 – 90%) nanoparticle. a) The interionic distance at which the expected number of ions equals one (illustrated in the inset) as a function of the doping concentration of Nd^3+^ ions. The dotted line represents the average interionic distance in the hexagonal phase of UCNPs

**
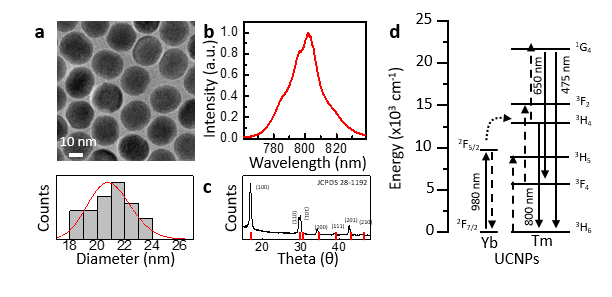
**

**Supplementary Figure 3.** Characterizations of UCNPs. a) TEM images and corresponding size distribution and pattern of XRD. b) NIR emission spectrum of UCNPs under 980 nm excitation. c) XRD patterns of UCNPs. d) 980-to-800 nm up-conversion process of UCNPs.


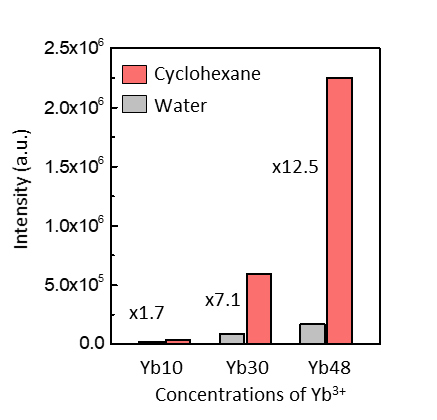


**Supplementary Figure 4.** Comparison of the 800 nm emission intensity of nanoparticles with different Yb^3+^ concentrations (10, 30 and 48%) under 980 nm excitation.


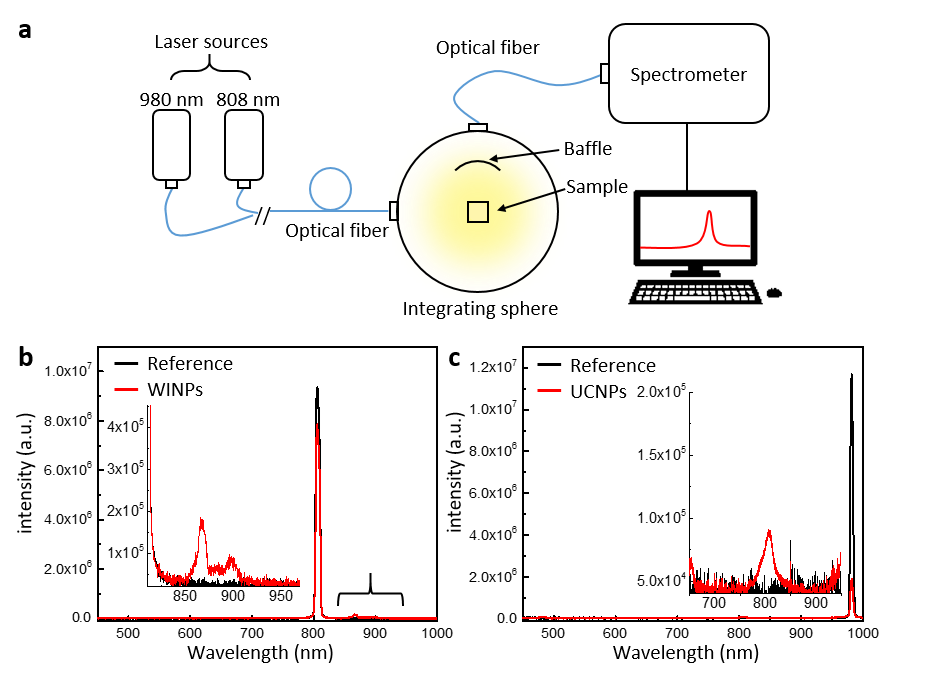


**Supplementary Figure 5.** Measurement of quantum yields. a) Schematic illustration of the setup used for quantum yield measurement. b) Measured excitation and emission power spectrum from the WINPs and the reference sample under 800 nm excitation (2 W cm^-2^). c) Measured excitation and emission power spectra from the UCNPs and the reference sample under 980 nm excitation (2 W cm^-2^). The reference sample consist of a cyclohexane solution without nanoparticles.


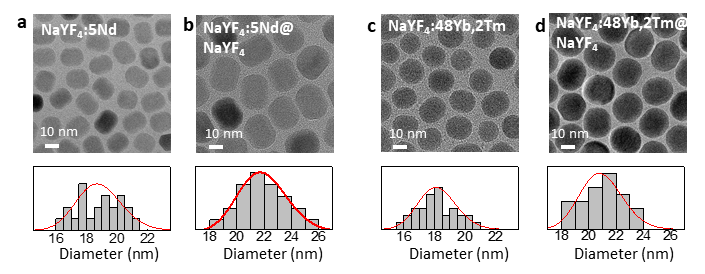


**Supplementary Figure 6.** Comparison of the 800 nm emission intensity of nanoparticles with different Yb ratios under 980 nm excitation


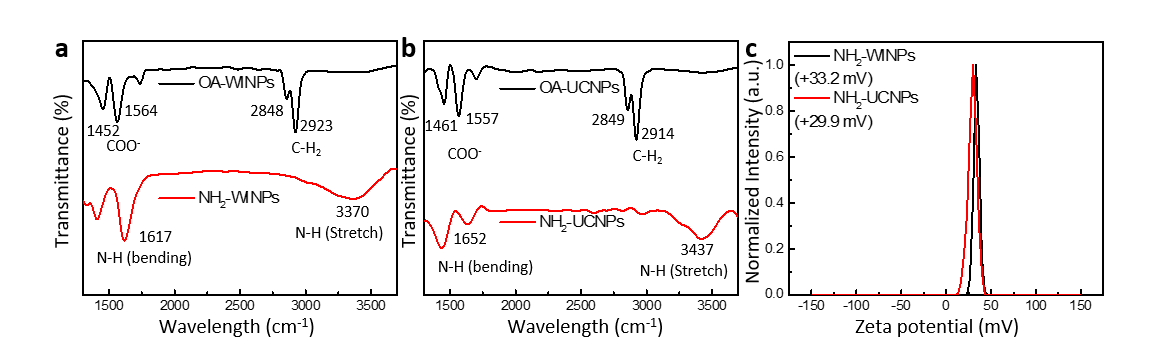


**Supplementary Figure 7**. Surface modifications of nanoparticles a) and b) Fourier transform infrared spectroscopic (FT-IR) spectrum of oleic acid (black) and dopamine-ligand (red). The surface modifications were confirmed by the appearance of two new bends at around 1600 and 3400 cm^-1^, attributed to N–H bending and stretching of amine group. c) Zeta potential profiles of NH_2_-ligand exchanged WINPs (+33.2 mV) and UCNPs (+29.9 mV).


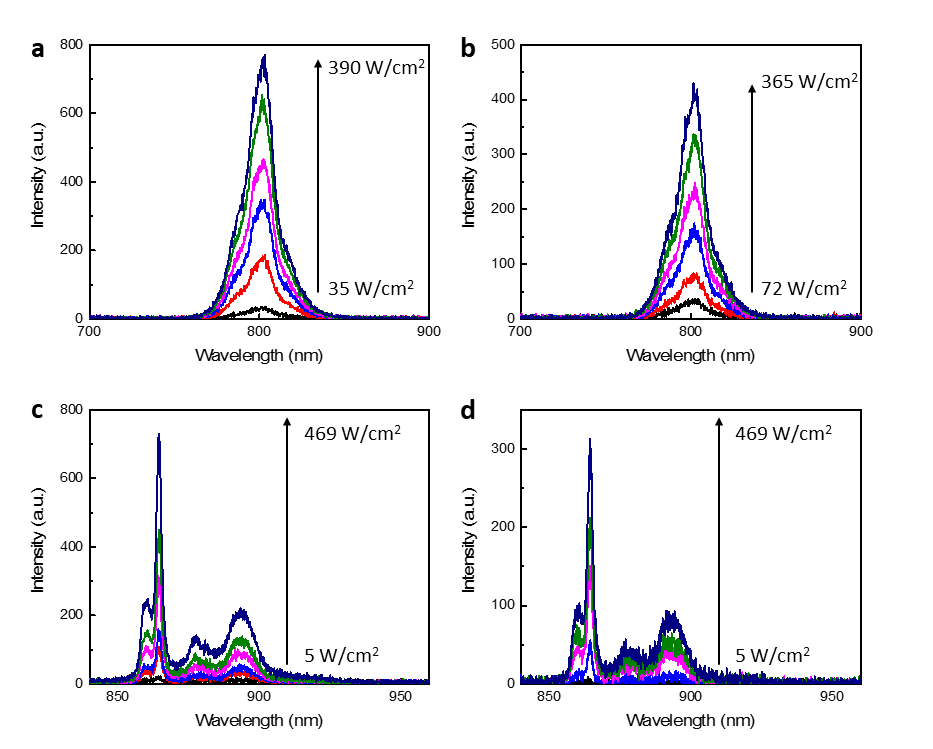


**Supplementary Figure 8.** NIR spectrum obtained from single-particle-level images a) UCNPs in dry conditions and b) in water conditions. c) WINPs in dry conditions and d) in water conditions.


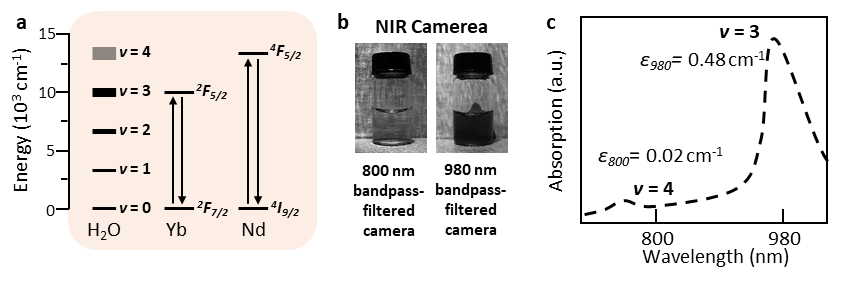


**Supplementary Figure 9.** NIR properties of water. a) absorption coefficient of water and Yb ions, and Nd ions. b) NIR optical images of water for c) NIR absorption spectrum of water.


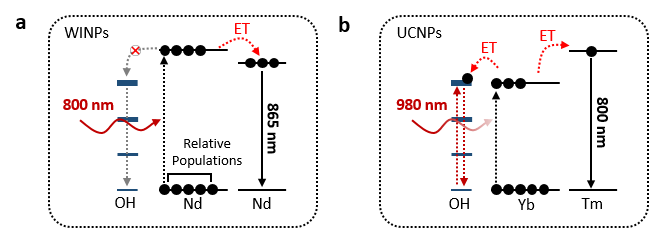


**Supplementary Figure 10.** a) Schematic illustrations of relative population and energy transfer models of the WINPs in water and, b) UCNPs in water.

**
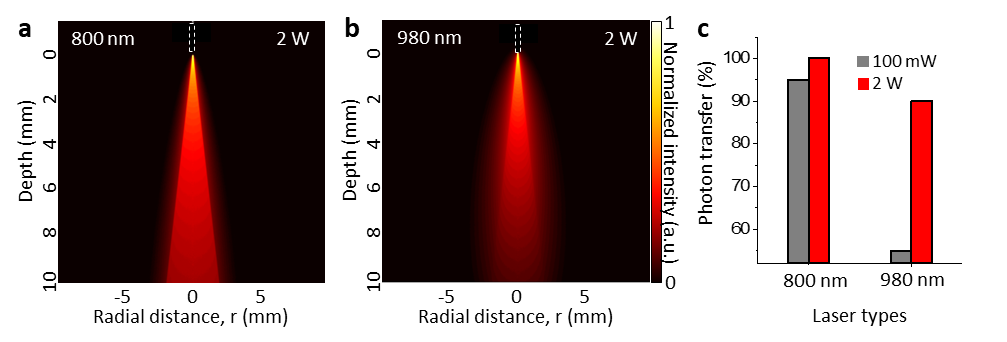
**

**Supplementary Figure 11.** Monte Carlo simulation of light propagation. a) and b) Heatmap profiles for light originating from the fiber (100 µm, NA = 0.22, g = 0.9, output power = 2 W) placed water solution via Monte Carlo simulation as a function of distance. c) Comparison of photon attenuation by laser type and output power.


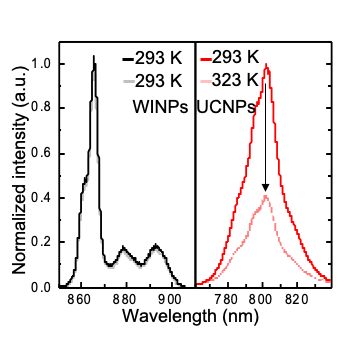


**Supplementary Figure 12**. Thermal induced emission quenching in NIR-I region of nanoparticles.


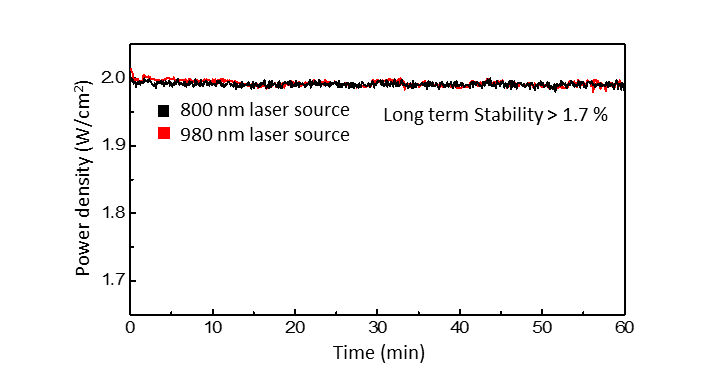


**Supplementary Figure 13**. Stability test of two types of lasers with 2 W output for1 hour


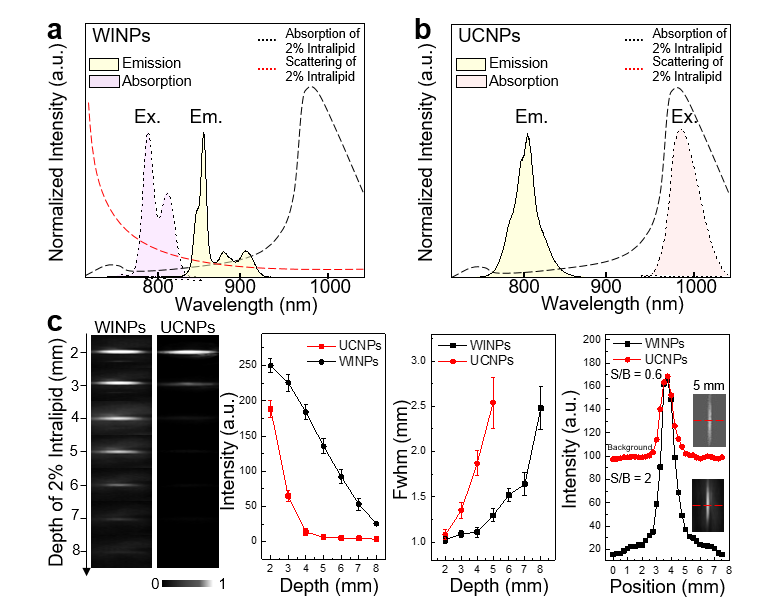


**Supplementary Figure 14**. Intralipid phantom characterizations in NIR-I window. a) and b) Scattering coefficient and absorption coefficient of 2 % intralipid solution in NIR-I window and absorption and emission spectrum of WINPs and UCNPs. c) NIR-I imaging in 2 % intralipid solution with different depths of 2 ~ 8 mm under 100 mW cm^-2^ irradiation.


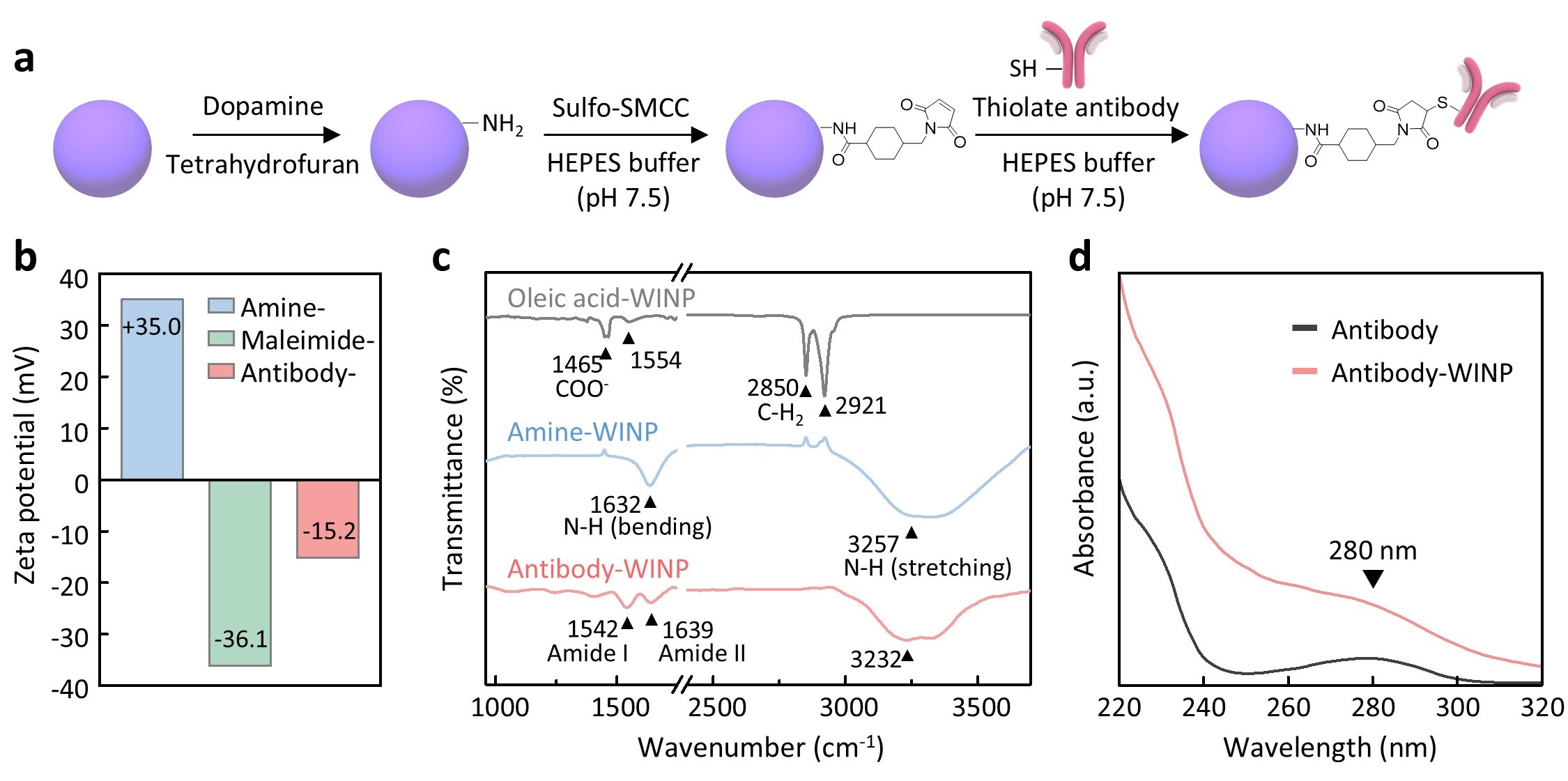
**Supplementary Figure 15.** Surface modification of the WINPs. a) Schematic illustration of conjugation path of anti-AIV detector antibody (Ab) and WINPs. b) Zeta potentials of amine-, maleimide-, and Ab-WINP. c) FT-IR spectra of oleic acid capped, amine-, and Ab-WINP. d) UV-vis spectra of anti-AIV detector Ab and Ab-WINP. The observed peak of 280 nm indicates the immobilization of antibody.


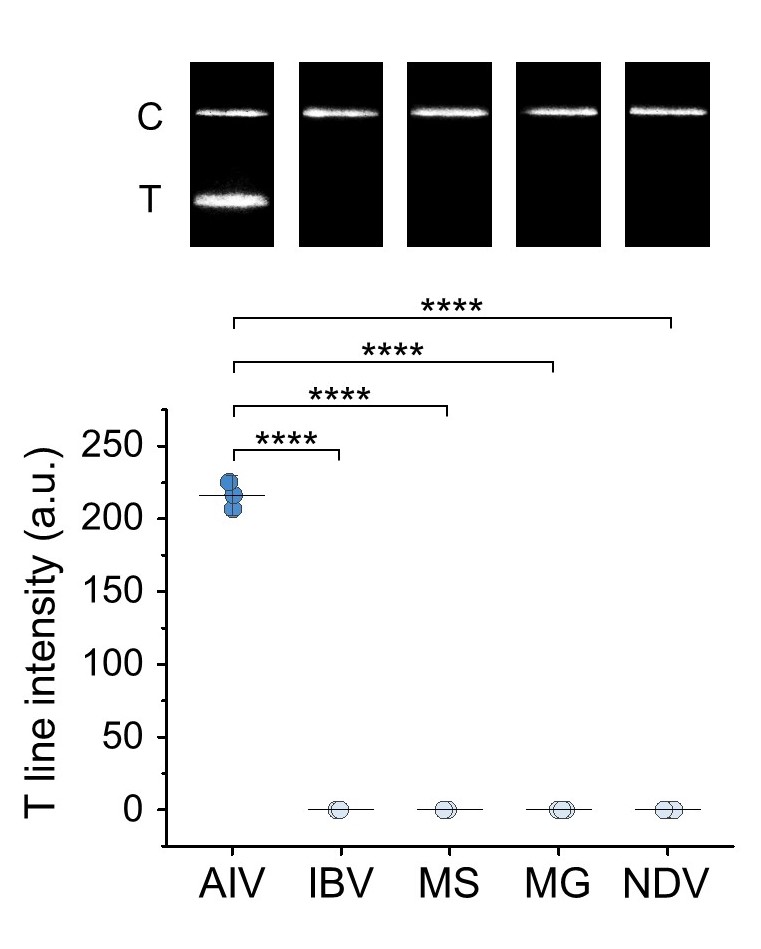


**Supplementary Figure 16.** Specificity test of WINPs-LFA. WINPs-LFA demonstrated specificity for AIV, with no cross-reactivity towards other avian pathogens, including infectious bronchitis virus (IBV), *Mycoplasma gallisepticum* (MG), *Mycoplasma synoviae* (MS), and Newcastle disease virus (NDV). The test line intensities were obtained from the 8-bit ImageJ-processed images of LFA strips. Data are presented as mean (*n*=3) values ± SD. Statistical analysis using a two-tailed unpaired t-test: “****” means *p* < 0.0001.


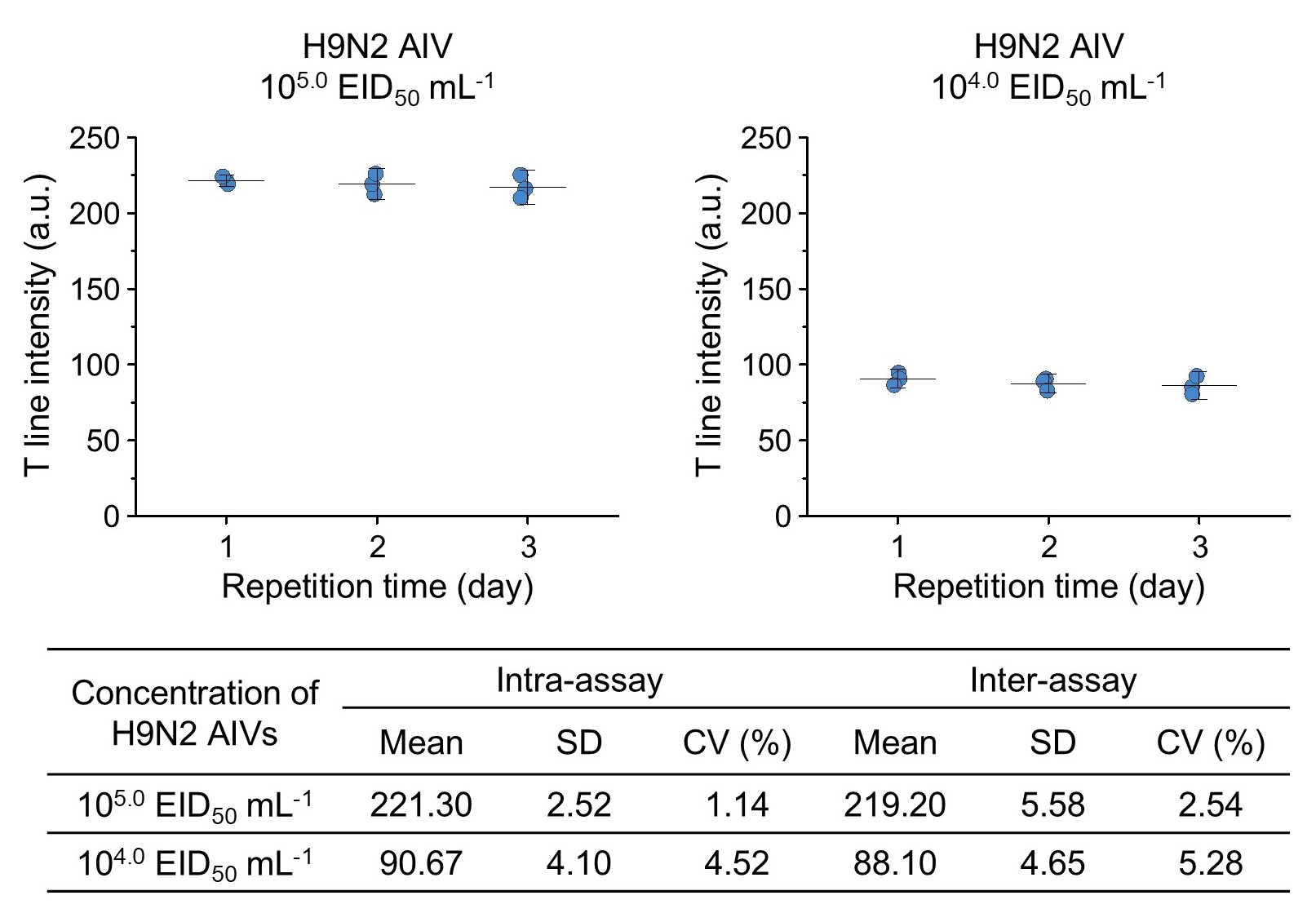


**Supplementary Figure 17.** Repeatability tests of the WINPs-LFA**.** The repeatability of the WINPs-LFA was evaluated based on intra-assay and inter-assay coefficients of variation (CVs). Test line intensities using H9N2 AIVs at concentrations of 10^5.0^ and 10^4.0^ EID_50_ mL^-1^ were measured three times a day for three consecutive days under 800 nm excitation (100 mW cm^-^²). The test line intensities were obtained from the 8-bit ImageJ-processed images of LFA strips. Data are presented as mean (*n*=3) values ± SD.


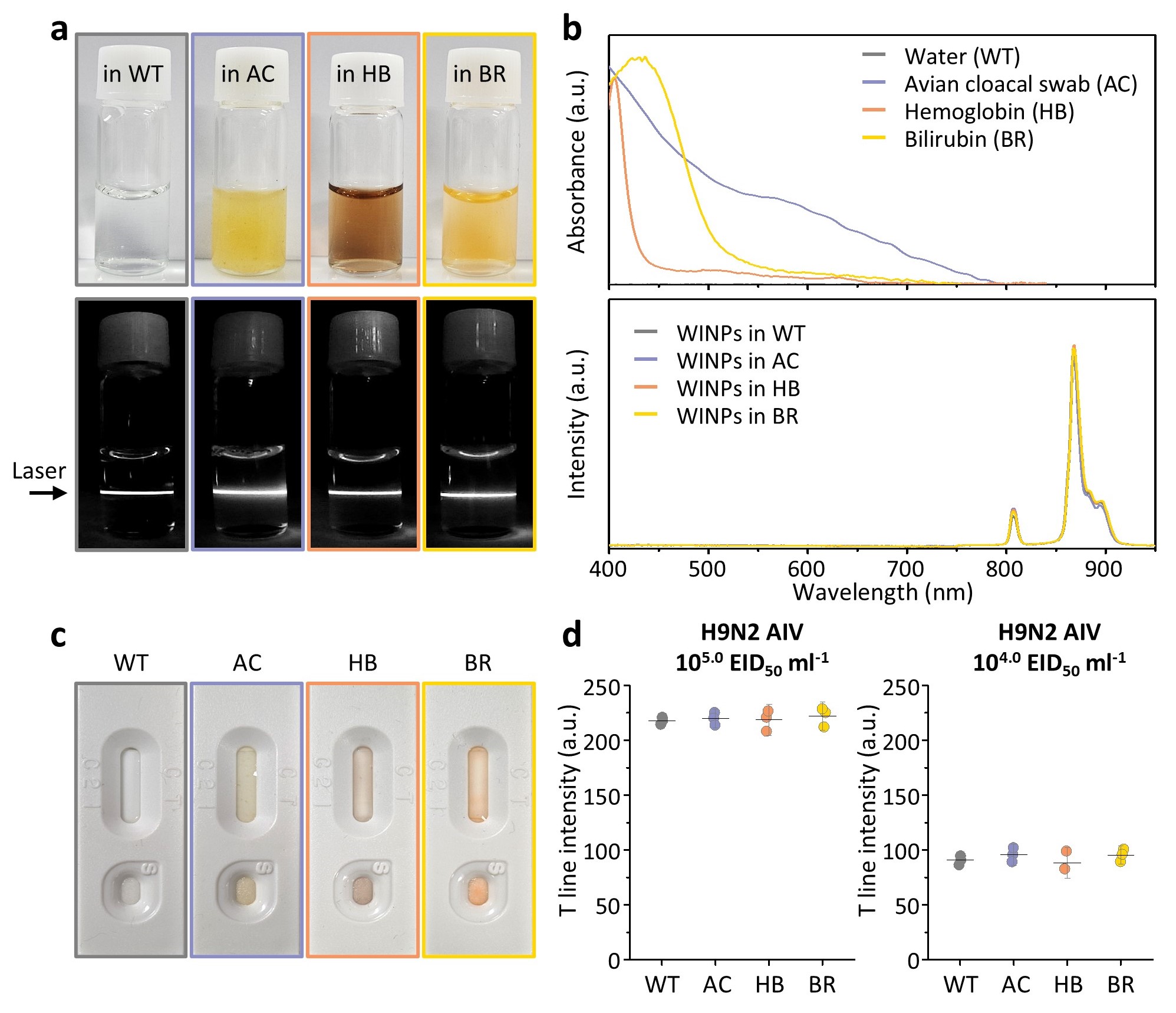


**Supplementary Figure 18.** Anti-interference ability of the WINPs**.** a) Photographic images of the WINPs dispersed in transparent water and colored biological solutions (avian cloacal swab, hemoglobin, bilirubin) under 800 nm excitation. The concentration of hemoglobin and bilirubin solutions is 2 mg mL^-1^. b) Absorbance spectra of the solutions and luminescence emission spectra of the WINPs dispersed in the solutions under 800 nm excitation (100 mW cm^-^²). c) Photographic images and d) T line intensities of WINPs-LFA after adding H9N2 AIVs (10^5.0^ and 10^4.0^ EID_50_ mL^-1^) diluted in water, avian cloacal swab, hemoglobin, and bilirubin. The test line intensities were obtained from the 8-bit ImageJ-processed images of LFA strips. Data are presented as mean (*n*=3) values ± SD.

**
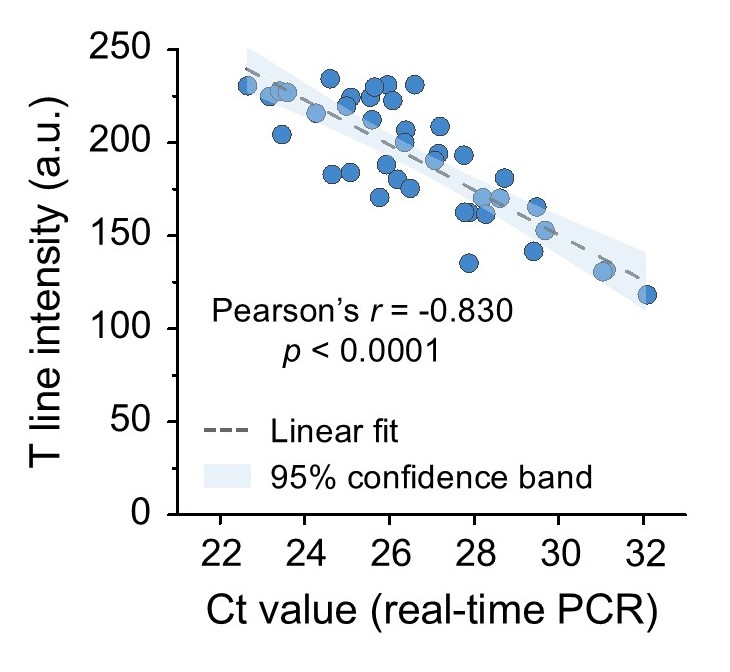
**

**Supplementary Figure 19.** Correlation between the WINPs-LFA and real-time PCR for AIV detection, showing high correlation with a Pearson’s coefficient (*r*) of -0.830. Statistical significance was tested by a two-tailed unpaired t-test. *r* < 0 indicates a negative correlation and 0.8 < | *r* | < 1 indicates a high correlation.

**Table S1.** Summary of optical properties of various down-shifting lanthanide-doped nanoparticles

| Lanthanide | Tm^3+^ | Er^3+^ | Yb^3+^ | Nd^3+^ |
| --- | --- | --- | --- | --- |
| Absorption | 785 nm  (^2^F_7/2_ 🡪 ^2^F_5/2_) | 790 nm  (^4^I_15/2_ 🡪 ^4^I_9/2_) | 960 – 980 nm  (^2^F_7/2_ 🡪 ^2^F_5/2_) | 790 – 810 nm  (^4^I_9/2_ 🡪 ^4^F_5/2_) |
| Absorption cross-section | 8.25 x 10^-22^ cm^2^ | 1.4 x 10^-21^ cm^2^ | 1.77 x 10^-20^ cm^2^ | 1.25 x 10^-19^ cm^2^ |
| Emission (NIR-I) | 800 nm (^3^H_4_ 🡪 ^3^H_6_) in NIR-I | X | X | 865 nm (^4^F_3/2_ 🡪 ^4^I_9/2_) in NIR-I |
| Emission (NIR-II) | 1450 nm  (^3^H_4_ 🡪 ^3^F_4_) in NIR-IIb | 1540 nm  (^4^I_13/2_ 🡪 ^4^I_15/2_) in NIR-IIb | 980 – 1020 nm  (^2^F_5/2_ 🡪 ^2^F_7/2_) in NIR-IIa | 1064 nm  (^4^F_3/2_ 🡪 ^4^I_11/2_) in NIR-IIa |
| Q.Y. | 9.07 ~ 14.16 % | 10.2 ± 0.8 % | NA | 22.1 ± 0.9 % |
| Ref | ^1-3^ | ^4^ | ^5^ | (This work)^5^ |

**Table S2.** Summary of the power density of NIR lasers used for analysis of UCNPs-LFAs as previously reported.

|  | Excitation (nm) | Emission (nm) | Power density of laser | Target | LOD | Ref |
| --- | --- | --- | --- | --- | --- | --- |
| NaYF_4_:Yb,Tm | 980 | 800 | 1000 mW | Foot-and-mouth disease virus | 2.1×10^2^  TCID_50_ mL^-1^ | ^6^ |
| NaYF_4_:Yb,Tm@NaYF_4_:Ca | 980 | 800 | 1000 mW cm^-2^ | H5N2, H5N6 AIV | 10^2^, 10^3.5^  EID_50_ mL^-1^ | ^7^ |
| NaYF_4_:Yb,Tm@NaYF_4_ | 980 | 800 | 300 mW | procalcitonin | 0.03 ng mL^-1^ | ^8^ |
| NaYF_4_:Yb,Tm@NaYF_4_ | 980 | 800 | 100 mW cm^-2^ | H9N2 AIV | 10^4.53^ EID_50_ mL^-1^ | This work |
| NaYF_4_:Nd@ NaYF_4_ | 800 | 865 | 100 mW cm^-2^ | H9N2 AIV | 10^3.52^ EID_50_ mL^-1^ |  |

**Table S3.** Comparison between real-time PCR and the WINPs-LFA for detection of AIVs in 65 clinical samples.

| Sample | Swab | Real-time PCR  Ct* value | WINPs- LFA | Sample | Swab | Real-time PCR  CT value | WINPs- LFA |
| --- | --- | --- | --- | --- | --- | --- | --- |
| P#1 | OP | 24.65 | **+** | P#34 | OP | 32.09 | **+** |
| P#2 | OP | 23.46 | **+** | P#35 | OP | 25.96 | **+** |
| P#3 | OP | 27.88 | **+** | P#36 | OP | 26.08 | **+** |
| P#4 | OP | 26.19 | **+** | P#37 | V | 25.65 | **+** |
| P#5 | OP | 25.08 | **+** | P#38 | OP | 28.28 | **+** |
| P#6 | OP | 23.17 | **+** | P#39 | OP | 27.07 | **+** |
| P#7 | OP | 24.6 | **+** | P#40 | OP | 27.76 | **+** |
| P#8 | OP | 26.6 | **+** | N#1 | CL | N/A | **–** |
| P#9 | OP | 26.39 | **+** | N#2 | CL | N/A | **–** |
| P#10 | OP | 25.55 | **+** | N#3 | CL | N/A | **–** |
| P#11 | OP | 31.12 | **+** | N#4 | CL | N/A | **–** |
| P#12 | OP | 28.72 | **+** | N#5 | CL | N/A | **–** |
| P#13 | OP | 31.04 | **+** | N#6 | CL | N/A | **–** |
| P#14 | OP | 29.48 | **+** | N#7 | CL | N/A | **–** |
| P#15 | OP | 25.77 | **+** | N#8 | CL | N/A | **–** |
| P#16 | OP | 29.41 | **+** | N#9 | CL | N/A | **–** |
| P#17 | OP | 27.16 | **+** | N#10 | CL | N/A | **–** |
| P#18 | OP | 28.2 | **+** | N#11 | CL | N/A | **–** |
| P#19 | OP | 26.36 | **+** | N#12 | CL | N/A | **–** |
| P#20 | OP | 22.65 | **+** | N#13 | CL | N/A | **–** |
| P#21 | OP | 29.68 | **+** | N#14 | CL | N/A | **–** |
| P#22 | OP | 24.27 | **+** | N#15 | CL | N/A | **–** |
| P#23 | OP | 25.09 | **+** | N#16 | CL | N/A | **–** |
| P#24 | OP | 23.41 | **+** | N#17 | CL | N/A | **–** |
| P#25 | OP | 27.2 | **+** | N#18 | CL | N/A | **–** |
| P#26 | V | 24.99 | **+** | N#19 | CL | N/A | **–** |
| P#27 | OP | 23.59 | **+** | N#20 | CL | N/A | **–** |
| P#28 | OP | 25.59 | **+** | N#21 | CL | N/A | **–** |
| P#29 | OP | 26.49 | **+** | N#22 | CL | N/A | **–** |
| P#30 | OP | 25.93 | **+** | N#23 | CL | N/A | **–** |
| P#31 | OP | 28.61 | **+** | N#24 | CL | N/A | **–** |
| P#32 | OP | 27.88 | **+** | N#25 | CL | N/A | **–** |
| P#33 | OP | 25.59 | **+** |  |  |  |  |

*The cycle threshold (Ct) value of real-time PCR

Abbreviations: OP, oropharyngeal swab sample; V, vent swab sample; CL, cloacal swab sample; N/A, not applicable.

Positive and negative are represented by the ‘+’ and ‘**–**’, respectively, as tested by WINPs-LFA.

**Table S4.** Summary of the power density of NIR lasers and detectors used for analysis of NIR-based LFAs as previously reported.

| Detection strategy | Materials | Ex. (nm) | Em. (nm) | Excitation power | Target | LOD | Detector | Ref |
| --- | --- | --- | --- | --- | --- | --- | --- | --- |
| Fluorescence | BBTD@PS | 680 | 960 | 1.6 W | AFP | 0.24 ng mL^-1^ | CMOS | ^9^ |
| Fluorescence | PbS QDs | 780 | 1294 | NA | NSE | 0.28 ng mL^-1^ | InGaAs | ^10^ |
| Fluorescence | NIR-II fluorescent NPs | 808 | 1046 | NA | SARS-CoV-2 N protein | 0.01 ng mL^-1^ | InGaAs | ^11^ |
| Fluorescence | Ag_2_Se@PS (NIR-II QD) | 808 | 1020 | 10 W cm^-2^ | CEA | 0.768 ng mL^–1^ | InGaAs | ^12^ |
| Photothermal | Ag@Au TNPs | 808 | NA | 1 W cm^-2^ | SARS-CoV-2 N protein | 0.05 ng mL^-1^ | Thermal camera | ^13^ |
| Photothermal | Janus Au_shell_-Fe_3_O_4_ NPs | 808 | NA | 3 W cm^-2^ | Influenza A virus | 2 pg mL^-1^ | Thermal camera | ^14^ |
| Photoluminescence | WINPs (NaYF_4_:5%Nd@NaYF_4_) | 808 | 865 | 100 mW cm^-2^ | H9N2 AIV | 10^3.52^ EID_50_ mL^-1^ | Silicon-based detector | This work |

Abbreviations: Ex., excitation; Em., emission; BBTD@PS, Benzobisthiadiazole (NIR-II dye) loaded polystyrene; AFP, alpha fetoprotein; QDs, quantum dots; NSE, neuron-specific enolase; InGaAs, indium gallium arsenide; CEA, Carcinoembryonic antigen; Ag@Au TNPs, Ag@Au core/shell triangular nanoplates; NA, not available

**References**

1. Chang, Y. et al. Bright Tm^3+^-based downshifting luminescence nanoprobe operating around 1800 nm for NIR-IIb and c bioimaging. *Nature Communications* **14**, 1079 (2023).

2. Maalej, O. et al. Visible to near-infrared down-shifting in Tm^3+^ doped fluoride glasses for solar cells efficiency enhancement. *Optical Materials* **60**, 235-239 (2016).

3. Kong, M. et al. Luminescence Lifetime–Based In Vivo Detection with Responsive Rare Earth–Dye Nanocomposite. *Small* **15**, 1904487 (2019).

4. Li, H. et al. Clearable Shortwave-Infrared-Emitting NaErF4 Nanoparticles for Noninvasive Dynamic Vascular Imaging. *Chemistry of Materials* **32**, 3365-3375 (2020).

5. Matulionyte, M. et al. The Coming of Age of Neodymium: Redefining Its Role in Rare Earth Doped Nanoparticles. *Chemical Reviews* **123**, 515-554 (2023).

6. Kim, S. et al. On-Site Remote Monitoring System with NIR Signal-Based Detection of Infectious Disease Virus in Opaque Salivary Samples. *ACS Sensors* **8**, 1299-1307 (2023).

7. Kim, J. et al. Rapid and background-free detection of avian influenza virus in opaque sample using NIR-to-NIR upconversion nanoparticle-based lateral flow immunoassay platform. *Biosensors and Bioelectronics* **112**, 209-215 (2018).

8. Ji, T.X. et al. Background-Free Chromatographic Detection of Sepsis Biomarker in Clinical Human Serum through Near-Infrared to Near-Infrared Upconversion Immunolabeling. *ACS Nano* **14**, 16864-16874 (2020).

9. Chen, R. et al. NIR-II emissive lateral flow immunoassay for accurate determination of tumor marker in hemolysis. *Sensors and Actuators B*: *Chemical* **328** (2021).

10. Ao, L.J. et al. Sensitive and simultaneous detection of multi-index lung cancer biomarkers by an NIR-II fluorescence lateral-flow immunoassay platform. *Chemical Engineering Journal* **436** (2022).

11. Hu, R.B. et al. Sensitively detecting antigen of SARS-CoV-2 by NIR-II fluorescent nanoparticles. *Nano Research* **15**, 7313-7319 (2022).

12. Deng, K. et al. NIR-II fluorescent Ag2Se polystyrene beads in a lateral flow immunoassay to detect biomarkers for breast cancer. *Microchimica Acta* **190**, 462 (2023).

13. Wang, K. et al. Near-Infrared Responsive Ag@Au Nanoplates with Exceptional Stability for Highly Sensitive Colorimetric and Photothermal Dual-Mode Lateral Flow Immunoassay. *Analytical Chemistry* (2024).

14. Wen, C.Y. et al. Dual-mode and multiplex lateral flow immunoassay: A powerful technique for simultaneous screening of respiratory viruses. *Biosensors and Bioelectronics* **271**, 117030 (2025).
